# Assessment of transport phenomena in catalyst effectiveness for chemical polyolefin recycling

**DOI:** 10.1038/s44286-024-00108-3

**Published:** 2024-08-28

**Authors:** Shibashish D. Jaydev, Antonio J. Martín, David Garcia, Katia Chikri, Javier Pérez-Ramírez

**Affiliations:** 1 Institute for Chemical and Bioengineering, Department of Chemistry and Applied Biosciences, ETH Zurich, Zurich, Switzerland; 2 Büchi, Uster, Switzerland

**Keywords:** Chemical engineering, Catalysis, Fluid dynamics, Characterization and analytical techniques

## Abstract

Since the dawn of agitated brewing in the Paleolithic era, effective mixing has enabled efficient reactions. Emerging catalytic chemical polyolefin recycling processes present unique challenges, considering that the polymer melt has a viscosity three orders of magnitude higher than that of honey. The lack of protocols to achieve effective mixing may have resulted in suboptimal catalyst effectiveness. In this study, we have tackled the hydrogenolysis of commercial-grade high-density polyethylene and polypropylene to show how different stirring strategies can create differences of up to 85% and 40% in catalyst effectiveness and selectivity, respectively. The reaction develops near the H_2_–melt interface, with the extension of the interface and access to catalyst particles the main performance drivers. Leveraging computational fluid dynamics simulations, we have identified a power number of 15,000–40,000 to maximize the catalyst effectiveness factor and optimize stirring parameters. This temperature- and pressure-independent model holds across a viscosity range of 1–1,000 Pa s. Temperature gradients may quickly become relevant for reactor scale-up.

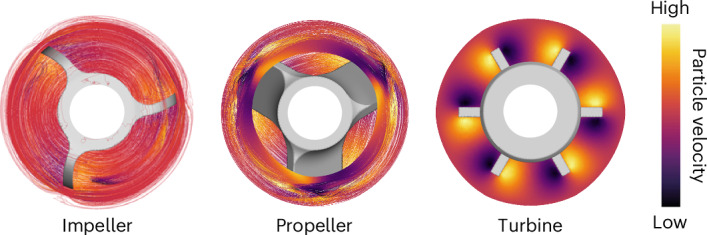

## Abstract (web summary)

The importance of optimizing the contact between catalyst particles, hydrogen and plastic melt in polyolefin chemical recycling has been overlooked, leading to suboptimal performance. The authors develop a criterion based on the dimensionless power number to optimize catalyst effectiveness. Stirring conditions can now be selected to treat commercial-grade polyethylene and polypropylene.

## Main

Some 12,000 years ago, our ancestors already possessed a rudimentary understanding of the benefits of agitation during brewing, when process efficiency was not a pressing concern^1^. The landscape changed dramatically with the inauguration of the chemical industry after the construction of the first soda ash plant in Widnes in 1847^2^. This marked the start of an era in which reducing costs and environmental pollution in large-scale processes became the imperative. Fast forward to the 20th century, pioneers such as G. Damköhler^3^, E. W. Thiele^4^ and J. J. Carberry^5,6^ conceptualized the notion of the ‘catalyst effectiveness’ and devised quantitative criteria to assess it in heterogeneously catalyzed reactions, integrating the concepts of catalyst design and reaction engineering. The catalytic chemical recycling of polyolefins, with the potential for processing more than 60% of global plastic waste^7^, is a prominent example of a catalyst design and reaction engineering challenge for contemporary chemical engineers^8^.

Focusing on the emerging hydrogenolysis strategy to tackle polyolefin waste, design efforts to find active catalytic surfaces offering control over the cleavage of polyolefins, which are more chemically resistant than functionalized polymers^9,10^, has steadily gained momentum over the past decade^11–18^. In contrast, reports on reaction engineering are scarce, despite distinctive features requiring careful attention, as extrapolation from already mature and related technologies, such as pyrolysis^19–22^, is not straightforward. The main reason is that the multiphasic operation, typically in batch mode, compounded by the presence of non-Newtonian polymer melts with viscosities exceeding that of honey by up to three orders of magnitude, can lead to highly ineffective mixing if technologies with high power per unit volume, such as mechanical stirring, are not used^23,24^. A recent report has raised awareness about transport phenomena in this field, exemplified by the application of classical diffusion theory to analyze external mass transport limitations in polyolefin depolymerization under equilibrium^25^. The inability of high-average-molecular-weight polyolefin chains to access the interior of porous catalyst particles has also been claimed to be a factor^26–28^, whereas for low-average-molecular-weight polyolefins, the accessibility of different types of plastic to the pores is mostly seen as a catalyst design strategy to tune selectivity^29,30^.

**Fig. 1:**
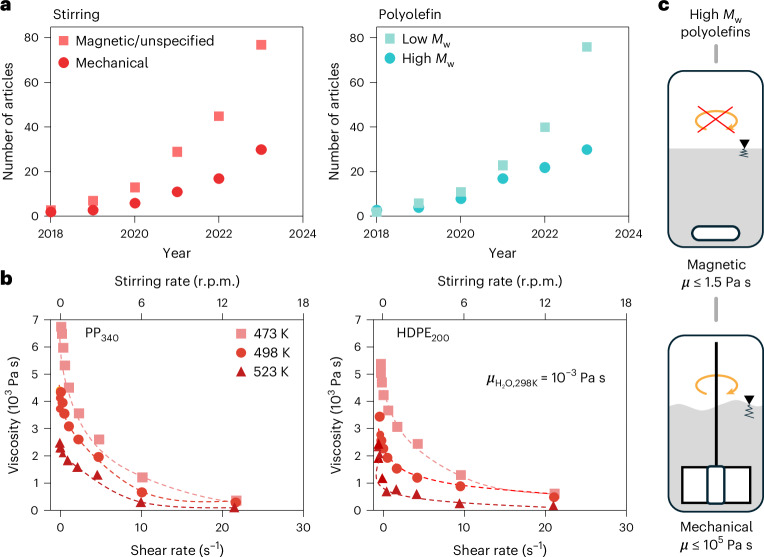
Literature analysis and influence of melt viscosity. **a**, Aggregated number of scientific publications on the hydrogenolysis/hydrocracking of polyolefins, classified according to the stirring configuration and molecular weight. Low *M*_w_ is defined as <100 kDa and high *M*_w_ is defined as >100 kDa. See Supplementary Table 2 for numerical values**. b**, Dependency of the viscosity of PP_340_ and HDPE_200_ on shear rate at different temperatures (Supplementary Tables 3 and 4). The approximate stirring rates required to reach the equivalent shear rates in a typical reactor used for the catalytic tests in this study are included. More details are available in Supplementary Note 1. The viscosity of water at 298 K is shown for comparison. **c**, Maximum viscosity of fluids amenable to the magnetic or mechanical stirrers usually available in research laboratories. High-molecular-weight (and some low-molecular-weight) plastics require mechanical stirring, as shown in Supplementary Videos 1 and 2. Source data

Increasing attention is directed toward low-molecular-weight plastics (<100 kDa, with limited commercial relevance) compared with consumer-grade plastics with molecular weights (*M*_w_) > 100 kDa. Magnetic (or often unspecified) stirring is, however, becoming the most popular mixing strategy regardless of the *M*_w_ of the studied plastic (Fig. 1a and Supplementary Tables 1 and 2). The lack of quantitative criteria regarding stirring in current testing protocols^31^ raises the question of the impact on catalyst effectiveness, with a lack of standardization and limited catalyst benchmarking, which are among the most prominent obstacles to the scaling up of catalytic technologies. Plastic recycling urgently needs modern chemical engineering tools to fully exploit catalyst design efforts.

Here we report quantitative guidelines for maximizing three-phase contact in this field of reaction engineering and demonstrate them for the hydrogenolysis of commercial-grade high-density polyethylene (HDPE) and polypropylene (PP). The guidelines are derived from a combination of experimental, theoretical and simulation studies, which led to a simple quantitative criterion based on the dimensionless power number to optimize catalyst effectiveness factors.

## Results

### Viscosity of molten polyolefins and stirring performance

The mixing of highly viscous substances (viscosity (*μ*) ≥ 10 Pa s)^23^ is mainly characterized by the difficulty in reaching turbulent flows due to high viscous energy dissipation leading to excessive power consumption and local hot spots in the reaction medium. Polymer melts are known to be non-Newtonian fluids with a viscosity dictated by shear rate (a measure of the rate at which parallel internal surfaces slide past one another; Extended Data Table 1) and temperature, potentially leading to local variations in the reactor that could affect the mixing regime during operation^32,33^. We selected HDPE (*M*_w_ = 200 kDa, denoted HDPE_200_) and PP (*M*_w_ = 340 kDa, denoted PP_340_) grades found in consumer goods such as plastic caps, cars or textiles, with the characteristic *M*_w_ used to label the polymers obtained from their melt flow index^34^.

We first conducted a rheological analysis, the results of which are shown in Fig. 1b (see Supplementary Tables 3 and 4 and Methods for details). As expected, increasing the shear rate led to a decrease in the viscosity at all of the tested temperatures, converging toward a distinctive value for each polymer (*~*500 Pa s for HDPE_200_ and ∼320 Pa s for PP_340_). Laminar flows with inherently low mixing capabilities are thus expected in polyolefin chemical recycling (Supplementary Note 1)^35^. Estimation of the stirring rates (*N*) required in a typical reactor vessel to reach the equivalent shear rates (secondary horizontal axis in Fig. 1b) revealed that temperature and local variations in viscosity in the melt under typical stirring rates can be disregarded (see Supplementary Note 1 for details).

The torque (*τ*) required to stir a non-Newtonian fluid is proportional to the product of its viscosity and a power law of the stirring rate, that is, *τ* ∝ *μN*^*α*^ (ref. ^36^), where *α* is an experimentally determined parameter dependent on the fluid. Taking into account that molten HDPE_200_ and PP_340_ have viscosities approximately one million times greater than that of water at room temperature ($\mu_{{\rm{H}}_2{\rm{O}},298{\rm{K}}}$ ≈ 0.001 Pa s), we verified that the magnetic stirrers commonly available in laboratories are incapable of stirring high-molecular-weight polyolefins and presumably do not allow good control over the stirring rate for low-molecular-weight ones, as illustrated in Fig. 1c and Supplementary Videos 1 and 2. Magnetic stirrers are suitable for viscosities lower than ~1.5 Pa s (ref. ^37^), whereas mechanical stirrers can be functional up to 10^5^ Pa s (ref. ^38^).

### Catalyst evaluation and computational fluid dynamics simulations

**Extended Data Fig. 1:**
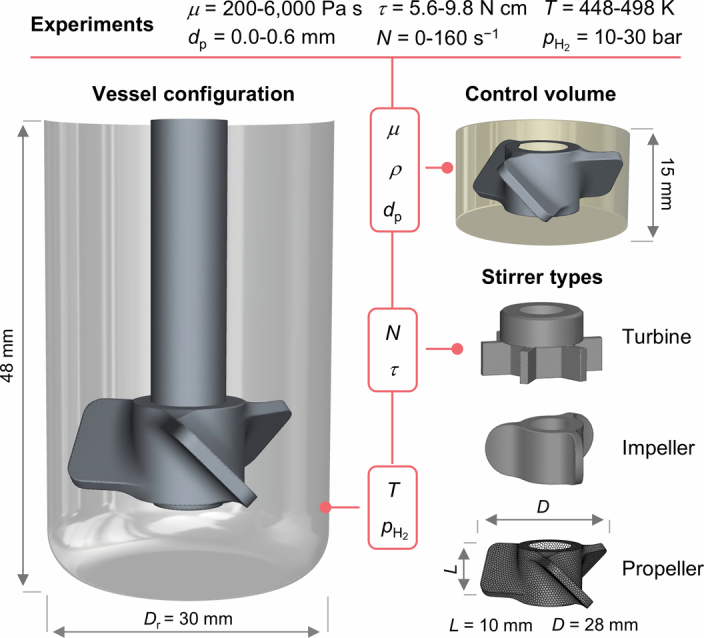
Geometry and reaction variables. Schematic representation of the main components of the testing setup (Supplementary Fig. 1) replicated in CFD simulations, including reactor vessel and stirrer types. Main dimensions are indicated, including those of the vessel, the height of the modelled polymer melt, and the diameter (*D*) and blade height (*L*) common to the three stirrer types. The mesh used for the propeller type in CFD simulations is shown as representative. The main experimentally imposed or monitored operation variables with their ranges are provided. Stirring of polyethylene with and without catalyst particles in the experimental setup is available in Supplementary Video 3.

Experiments were developed in a four-parallel reactor set-up (see Methods, Supplementary Fig. 1 and also Supplementary Video 3 for the stirring configuration). Ruthenium nanoparticles supported on titania, a state-of-the-art catalyst for the conversion of HDPE_200_, was used throughout the study (see Supplementary Fig. 2 for the characterization of the Ru/TiO_2_ catalyst)^39^. The factors driving the performance (listed in Extended Data Fig. 1) within the experimental limitations described in Supplementary Note 2 were systematically varied and translated into computational fluid dynamics (CFD) simulations, including experimentally obtained viscosity dependencies, to describe the hydrogen–catalyst–melt contact over time (see Methods for a general description, Supplementary Note 3 for scope and Supplementary Fig. 3 for convergence plots).

**Extended Data Fig. 2:**
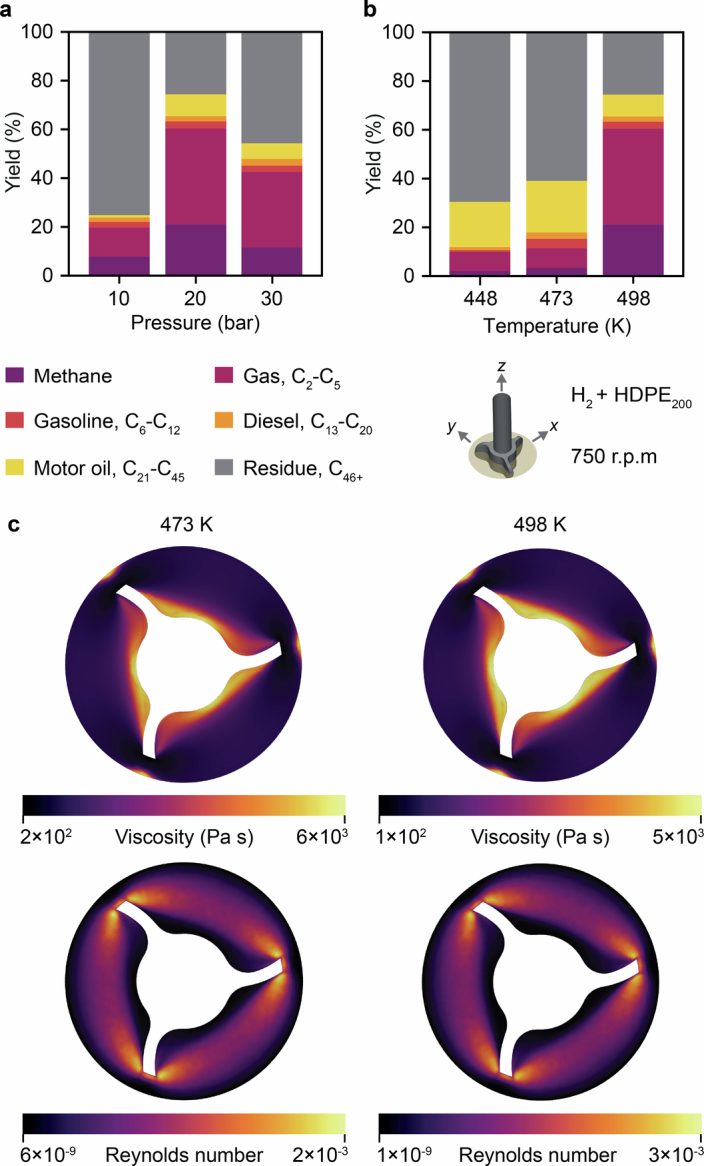
Influence of pressure and temperature on performance and viscosity. **a,b**, Variation of product distribution with pressure (**a**) and temperature (**b**) for hydrogenolysis of HDPE_200_. Corresponding results for PP_340_ are available in Supplementary Fig. 4. **c**, Three-phase CFD simulations of viscosity distribution (top) and Reynolds number (bottom) at the mid *x*-*y* plane of the stirrer at different temperatures. Values in Supplementary Table 7. Reaction and simulated conditions: *d*_p_ = 0.0-0.2 mm (0.2 mm for simulations), stirrer = impeller, stirring rate = 750 r.p.m. Source data

Initial experiments revealed optimum hydrogen pressures for HDPE_200_ and PP_340_ in accordance with the literature, with a pressure of 20 bar selected for subsequent tests (Extended Data Fig. 2, Supplementary Fig. 4 and Supplementary Tables 5 and 6)^40,41^, likely due to competitive adsorption of the polyolefin and hydrogen on the metal surface (Supplementary Fig. 5). Simulations at different temperatures confirmed minimum differences in the distribution of viscosities and Reynolds numbers (Extended Data Fig. 1 and Supplementary Table 7), in line with the observations depicted in Fig. 1b showing viscosity values almost independent of temperature at shear rates equivalent to stirring rates larger than approximately 15 r.p.m. in laboratory reactors (Supplementary Note 1). A commonly reported reaction temperature (498 K) was thus chosen for further analyses after performance tests (Extended Data Fig. 1)^42,43^.

### Internal and external mass transport limitations

**Fig. 2:**
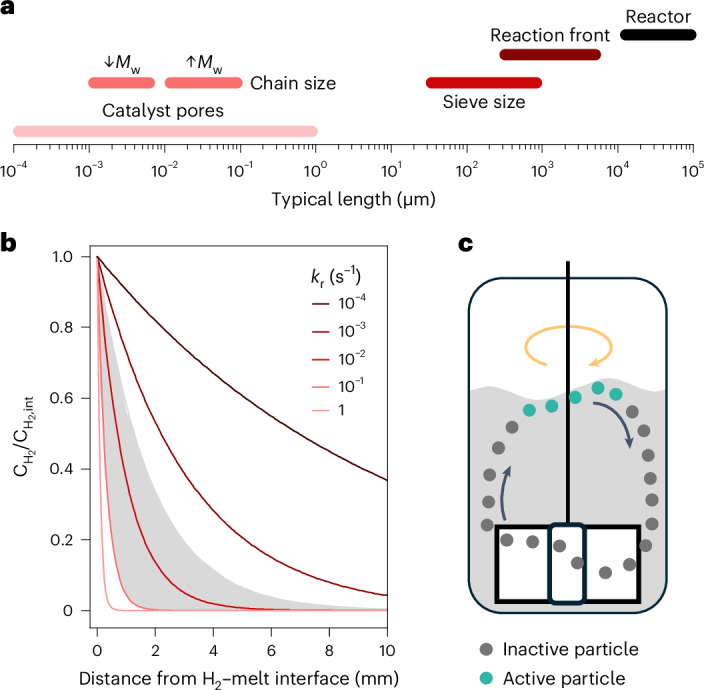
Characteristic lengths in catalytic polyolefin hydrogenolysis. **a**, Typical magnitudes of relevant length scales in the catalytic processing of consumer-grade plastics. Chain size refers to the typical dimensions of folded chains in low- and high-molecular-weight polymers. Reaction front refers to the characteristic penetration length of hydrogen in the melt before its concentration drops below 10% of its value at the H_2_-melt interface. **b**, Simulated decay of relative hydrogen concentration with distance from the H_2_–melt interface for different reaction rate constants (*k*_r_). ${\rm{C}}_{{{\rm{H}}_2},{\rm{int}}}$ refers to the concentration of H_2_ at the H_2_-melt interface. Pseudo-first-order kinetics ($r=k_{\rm{r}}c_{{\rm{H}}_2}$) were considered, as explained in more detail in Supplementary Note 5. The area shaded in gray indicates the typical range of *k*_r_ values calculated in our experiments and reported in the literature. **c**, Schematic representation of the circulation of catalyst particles in the reaction vessel, with those exposed to hydrogen and polymer melt shown in green as active particles toward hydrogenolysis, as deduced from **b**, highlighting the fact that the reaction is mostly constrained to the vicinity of the H_2_–melt interface. Source data

We first evaluated the ability of polymer chains to penetrate micropores and mesopores (see Supplementary Note 4 for the case of micrometer-sized pores usually found in shaped catalysts). The Freely Jointed Chain model (Supplementary Note 5) predicts a typical dimension for the folded chain (*Λ*) of ~ 22 nm for HDPE_200_^44–46^. For chain lengths below *Λ*, polymer chains tend to gradually favor the linear conformation^47^. The relevant pore size that polyolefins, or their liquid products following reaction, may not be able to penetrate thus ranges from *Λ* ≈ 1 nm (C_6_) to *Λ* ≈ 100 nm (high-molecular-weight polyethylene, *M*_w_ ≈ 5,000 kDa). These scales are represented in Fig. 2a and suggest that internal mass transport limitations in the case of porous catalysts may increase in relevance even for polyolefins with very high *M*_w_ as the reaction progresses toward shorter chain products, and therefore further studies are required. Internal mass transport phenomena were disregarded for simplicity as the Ru/TiO_2_ used in our study showed an average pore size of 6 nm within a very low specific pore volume of 0.02 cm^3^ g^−1^. Experiments at different stirring rates using a particle diameter of 0.6 mm support this assumption (Supplementary Fig. 6).

Regarding external mass transport limitations, an earlier study determined negligible external hydrogen gradients to catalyst particles immersed in the melt if equilibrium bulk concentrations of H_2_ are reached^25^. However, the simulation of H_2_ diffusion into molten HDPE_200_ in the absence of reaction for a range of hydrogen pressures, times and viscosities (Supplementary Fig. 7 and Supplementary Notes 6 and 7) revealed a characteristic time for equilibration beyond typically reported reaction times. In view of this, we computed the decay of the H_2_ concentration at the H_2_–melt interface assuming the direct reaction of H_2_ with the melt after estimating that the observed reaction rate is around five times that of the diffusion rate of H_2_, as provided by the Hatta number (Extended Data Table 2 and Supplementary Note 7)^48^. Figure 2b shows the results for different reaction rate constants (*k*_r_), defined according to the expression for pseudo-first-order kinetics, $r=k_{\rm{r}}c_{{\rm{H}}_2}$, where *r* is the rate of the reaction and $c_{{\rm{H}}_2}$ is the concentration of H_2_. A suitable range of *k*_r_ was estimated from the typical hydrogen consumption and reaction times observed in our study and reported in the literature (see Supplementary Note 7 for details)^30^. Poorly active catalysts not yielding any liquid products are characterized by *k*_r_ values of ∼1.5 × 10^−3^ s^−1^, whereas highly active systems able to provide 100% conversion into methane are expected to present *k*_r_ values of ∼0.1 s^−1^. These values translate into a range of concentration decays, highlighted in gray in Fig. 2b. Typically, we obtained values for *k*_r_ of ∼0.01 s^−1^. As observed in Fig. 2b, the concentration of hydrogen drops below 10% of the interface value within a few millimeters in all cases, strongly suggesting that the reaction is mostly confined to the vicinity of the H_2_–melt interface with a typical length (*λ*) of ∼10^−3^ m, leaving most of the melt non-reactive (Fig. 2a). A representation in which only catalyst particles exposed to this region are active toward the reaction (Fig. 2c) is thus a suitable approximation to study the role of agitation in catalytic performance.

### Impact of catalyst particle circulation on performance

The previous analysis shows the benefit of stirring configurations maximizing the presence of catalyst particles in the vicinity of the H_2_–melt interface. A first analysis based on the ratio of gravitational and viscous forces given by the Archimedes number (Ar; Extended Data Table 2) predicted Ar = 10^−8^–10^−7^ and therefore that the density of the catalyst is expected to play a negligible role in particle motion (Supplementary Note 8). Nevertheless, the average catalyst particle diameter (*d*_p_) is important as it determines the tendency of particles to follow melt streamlines according to the Stokes number (Stk; Extended Data Table 2)^49^. Considering *λ* as the characteristic length, Stk ≈ 10^−2^–10^0^ for *d*_p_ = 10^−4^–10^−3^ m. These values indicate that small catalyst particles will closely follow streamlines, whereas larger ones may deviate from them to an extent comparable to *λ*.

**Fig. 3:**
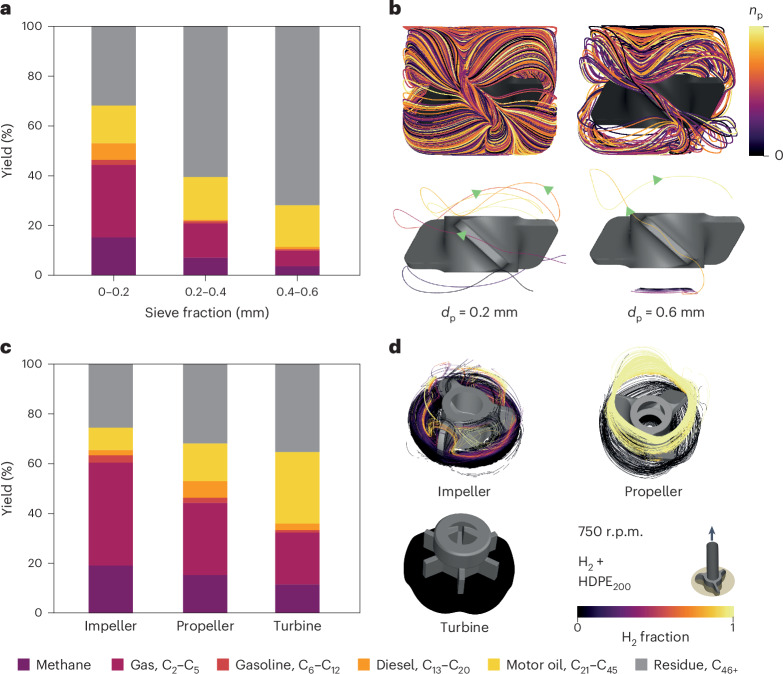
Influence of catalyst particle motion on performance. **a**, Variation in the product distribution for the hydrogenolysis of HDPE_200_ with catalyst sieve fraction using a propeller stirrer after 4 h. **b**, Corresponding three-phase CFD simulations using discrete phase modeling showing the trajectories of 0.2 and 0.6 mm catalyst particles under steady-state conditions: the total number of particles (*n*_p_, with *n*_p_ = 196 for 0.2 mm and *n*_p_ = 58 for 0.6 mm; top) and representative initial trajectories of individual particles (bottom). **c**, Variation in the product distribution for the hydrogenolysis of HDPE_200_ with stirrer type after 4 h. **d**, Corresponding CFD simulations (top views) of catalyst particle trajectories for different stirrers, colored according to the H_2_ fraction in the vicinity. Simulations for other sieve fractions and parallel analyses for PP_340_ can be found in Supplementary Figs. 8–10 and Supplementary Tables 5–7. Simulated particle trajectories are presented in Supplementary Video 4. Reaction and simulation conditions: *T* = 498 K, ${p}_{{{\rm{H}}}_{2}}$ = 20 bar, catalyst/plastic ratio = 0.05 and stirring rate = 750 r.p.m. Source data

We evaluated the importance of *d*_p_ by comparing the catalytic performance of three different catalyst sieve fractions (0–0.2, 0.2–0.4 and 0.4–0.6 mm) in the hydrogenolysis of HDPE_200_ (Fig. 3a and Supplementary Tables 5 and 6). Equivalent experiments with PP_340_ did not show substantial variation in the total yield due to its low reactivity effectively limiting the performance of the catalyst (Supplementary Fig. 8). However, the same trend was confirmed by using the shorter and thus more reactive PP_12_ (Supplementary Table 8 and Supplementary Fig. 9). We found the smallest sieve fraction to be beneficial, producing a 40% greater yield of the C_1_–C_45_ products compared with the largest sieve fraction under the tested conditions. CFD simulations for *d*_p_ = 0.2, 0.4 and 0.6 mm, keeping the same stirrer geometry, predicted differences in the particle trajectories. Larger particles on average required longer times to leave the bottom of the reactor and tended toward a more irregular occupancy of the vessel volume (Fig. 3b and Supplementary Fig. 10 for the three modeled particle sizes). Having determined the benefits of smaller sieve fractions, *d*_p_ = 0.2 mm was used for the rest of the simulations in this study.

**Extended Data Fig. 3:**
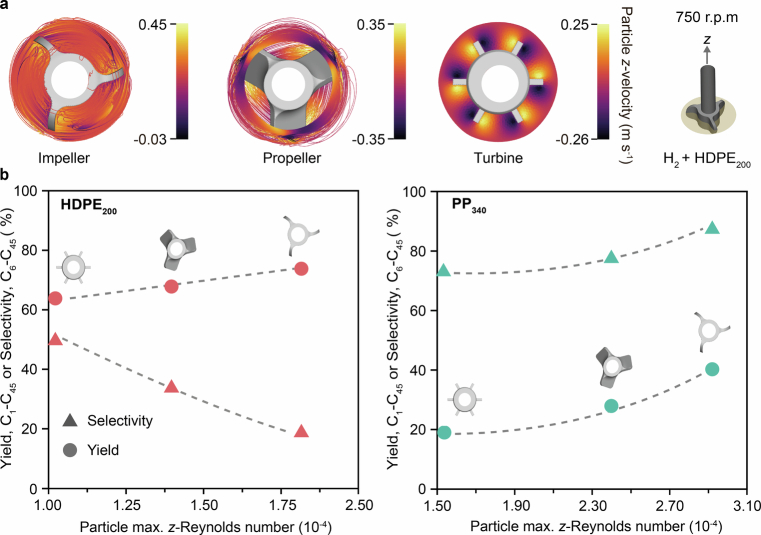
Influence of vertical motion of catalyst particles in performance. **a**, Three-phase CFD simulated distributions using discrete phase modelling from a top view of catalyst particle velocity along the *z*-axis. **b**, Correlation between total yield to C_1_-C_45_ products and selectivity to liquid products (C_6_-C_45_) with average particle *z*-Reynolds number for HDPE_200_ and PP_340_. Simulated particle trajectories are available in Supplementary Video 4. Reaction and simulated conditions: *T* = 498 K, *p*_H2_ = 20 bar, catalysts/plastic = 0.05, stirring rate = 750 r.p.m. Source data

**Extended Data Fig. 4:**
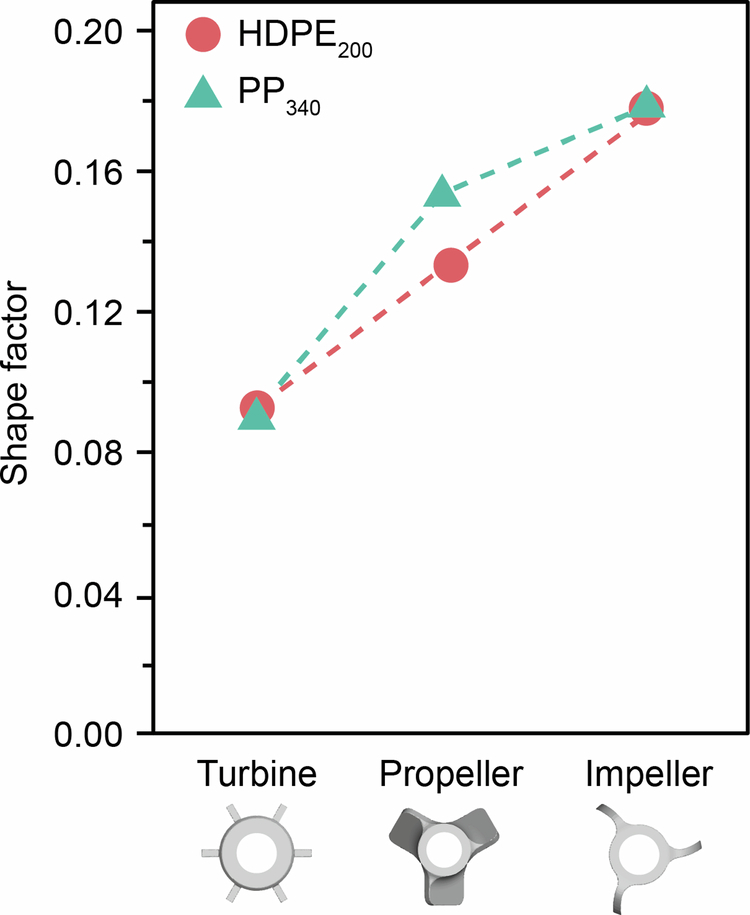
Shape factor. Variation of the shape factor (*K*_s_) defined as the ratio of the maximum vertical velocity of catalyst particles and velocity of the tip of the stirrer, determined via CFD simulations, for three different stirrer types. Values can be found in Supplementary Table 8. Source data

The stirrer imposes the flow pattern that catalyst particles follow (Supplementary Fig. 11). Figure 3c shows the product distribution for the catalytic hydrogenolysis of HDPE_200_ using three different stirrer geometries under the same conditions (Supplementary Tables 5 and 6 and Supplementary Fig. 12 for PP_340_). The yield of C_1_–C_45_ products was not greatly affected by the stirrer type, whereas the product distribution shifted from gas to liquid fractions, with the amount of gaseous product decreasing in the order impeller > propeller > turbine, highlighting that stirring strategies can tune selectivity, as a consequence of tuning the activity, given that the hydrogenolysis is a series of reactions, and must be reported to facilitate benchmarking. The total number of carbon–carbon bonds followed the same trend, as determined using the recently published procedure for calculating the number of backbone scission, isomerization and demethylation events^15^ (Supplementary Table 9). Figure 3d and Supplementary Video 4 show critical differences between the stirrers. Propellers tend to split the catalyst particles into two separate zones with either high or low H_2_ concentration. Impellers tend to keep catalyst particles circulating around the mid plane, where the H_2_ concentration is high due to the V shape adopted by the H_2_–melt interface. Impellers are thus better suited to optimizing catalyst use. The turbine is poorly efficient in transferring particles to H_2_-rich zones, leading to the modest generation of gaseous products (which require more molecules of H_2_ per molecule of polymer). These effects can be quantitatively understood by considering the maximum value of the vertical component of the particle Reynolds number (Re_p,*z*,max_; Extended Data Figs. 3 and 4, Supplementary Notes 9 and 10, Supplementary Tables 7,10 and 11, and Supplementary Fig. 13) as the first performance descriptor. Re_p,*z*,max_ can be derived from the melt properties, stirring rate, and particle and stirrer geometries and can be linked to activity and therefore changes in selectivity (Extended Data Fig. 2), offering a first tool to predict performance trends.

### Criterion to maximize the catalyst effectiveness factor

**Fig. 4:**
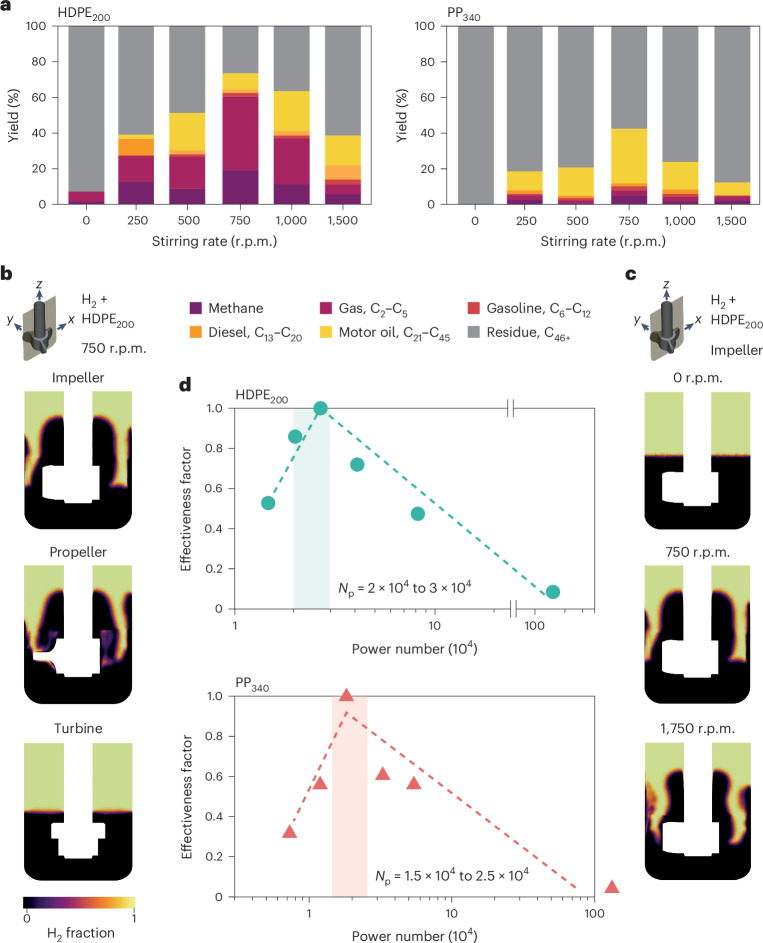
Criterion for maximizing the effectiveness factor. **a**, Variation in the product distribution with stirring rates for HDPE_200_ and PP_340_ with the impeller stirrer. **b**,**c**, Two-phase CFD simulations of the hydrogen fraction in the mid *z*–*x* plane for different stirrer types (**b**) and different stirring rates for the impeller stirrer (**c**). **d**, Correlation between the effectiveness factor, defined as the ratio between the yield of C_1_–C_45_ and maximum yield of C_1_–C_45_ in **a**, and the modified power number for HDPE_200_ and PP_340_, calculated using the stirring rates in **a** and the simulated fraction of H_2_ in Extended Data Fig. 5. Reaction and simulation conditions: *T* = 498 K, ${p}_{{{\rm{H}}}_{2}}$ = 20 bar and catalyst/plastic ratio = 0.05. Source data

The yield of C_1_–C_45_ products did not monotonically increase with stirring rate, as shown in the catalytic tests for both polymers (Fig. 4a). The existence of an optimum rate, in accord with some reports in the literature on the conversion of low-molecular-weight plastics^50,51^, led us to study the influence of stirring rate on the extent of the H_2_–melt interface. CFD simulations developed for the three stirrer types (Fig. 4b) predicted the potential of propellers and impellers to increase the interface. The ability of simulations to reproduce the V shape of the H_2_–melt interface for highly viscous plastics is confirmed in Supplementary Video 3. CFD simulations of the impeller at different stirring rates strongly hint at a relationship between stirring rate and the H_2_–melt interfacial area (Fig. 4c). Small variations in the distance between the base of the stirrer and the bottom of the vessel also led to small changes in the H_2_–melt interface. However, an excessive distance (the top of the stirrer at the free melt surface) led to a decrease in the interface (Supplementary Fig. 14). In general, the shear-thinning character of molten plastics makes stirring only effective in the imaginary volume occupied by the stirrer under rotation or slightly beyond, making it advisable to minimize the distance between the stirrer and reactor walls.

Given the difficulty of calculating the extent of the H_2_–melt interface, we defined as a proxy the fraction of hydrogen ($\chi_{{\rm{H}}_2}$; Extended Data Table 2) in a volume contained between the bottom of the stirrer and the H_2_–melt interface (Extended Data Fig. 1) when there is no stirring. The average Reynolds number in this region could serve as a descriptor for $\chi_{{\rm{H}}_2}$ as more turbulence (larger Re values) may lead to more pronounced hills and valleys on the surface of the melt. However, Re is not observable. For the case of Re ≪ 1 as studied here, the power and Reynolds number are inversely linearly correlated. The power number (*N*_p_) expresses the relationship between resistance and inertia forces and can be written in terms of $\chi_{{\rm{H}}_2}$ and observable variables such as the average density of the melt (${\bar{\rho }}$), the reactor diameter (*D*) and the average density of the melt (*ρ*_m_) (equation (1), Extended Data Table 2 and Supplementary Note 11)^24,52^.

1 $$\begin{array}{l}{N}_{{\rm{p}}}=\\\displaystyle\frac{2\uppi N\tau }{60\bar{\rho }(N/60)^{3}{D}^{5}}=\displaystyle\frac{7200\uppi \tau }{[\,{\chi }_{{\rm{H}}_2}{\rho }_{{\rm{H}}_2}+(1-{\chi }_{{\rm{H}}_2}){\rho }_{{\rm{m}}}]{N}^{\,2}{D}^{5}}\approx \displaystyle\frac{7200\uppi \tau }{(1-{\chi }_{{\rm{H}}_2}){\rho }_{{\rm{m}}}{N}^{\,2}{D}^{5}}\end{array}$$

**Extended Data Fig. 5:**
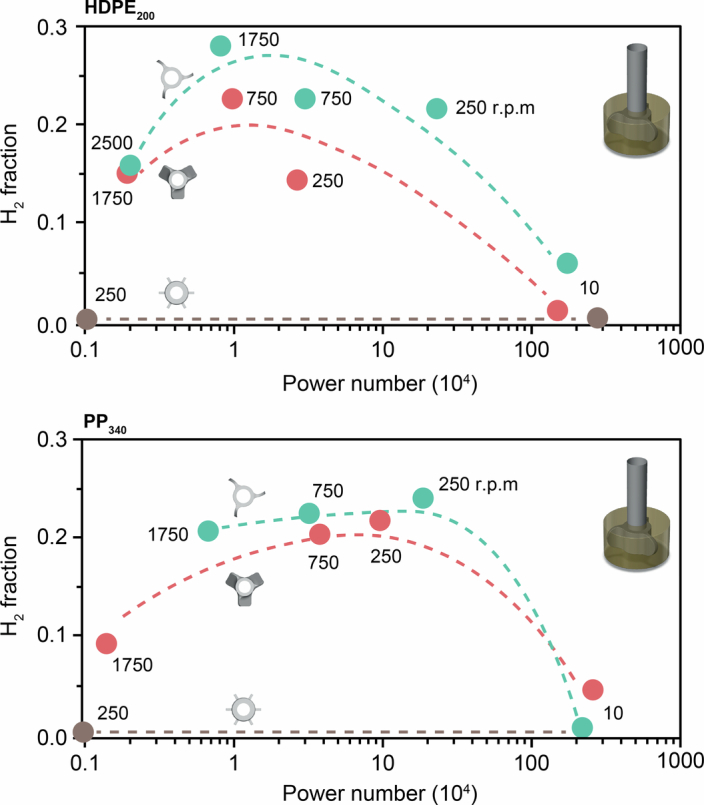
Simulated hydrogen fractions. Variation of the hydrogen fraction in the melt volume with power number at different stirring rates calculated from CFD simulations corresponding to those shown in Fig. 5 for the three stirrer types for HDPE_200_ (top) and PP_340_ (bottom). Values can be found in Supplementary Table 10. Source data

Extended Data Fig. 5 shows the relationship between $\chi_{{\rm{H}}_2}$ and *N*_p_ obtained from CFD simulations for HDPE_200_ and PP_340_ under the same conditions as used in Fig. 4a. Propeller and impeller stirrers yielded volcano behavior, with a maximum $\chi_{{\rm{H}}_2}$ ≈ 0.20–0.30 for *N*_p_ ≈ 10^4^–10^5^, shifted toward slightly lower values in the case for PP_340_. The difference in the optimal rates in Fig. 4a and Extended Data Fig. 5 can be ascribed to the lower average viscosity in reaction compared with the simulations (Supplementary Note 12), although in practice this has a small impact as $\chi_{{\rm{H}}_2}$ displays values of around 0.2 for a broad range of stirring rates.

Plots of the effectiveness factor (*η*), defined as the ratio between the yield of C_1_–C_45_ products and the maximum yield of C_1_–C_45_ products over a series of experiments (equation (2) and Extended Data Table 1), versus the corresponding *N*_p_ values based on the results presented in Fig. 4a show the optimal *N*_p_ ranges for the two polymers ($N_{{\rm{p}},{\rm{HDPE}}_{200}}$ ≈ 2 × 10^4^ to 3 × 10^4^ and $N_{{\rm{p}},{\rm{PP}}_{340}}$ ≈ 1.5 × 10^4^ to 2.5 × 10^4^) to achieve high *η* values (Fig. 4d) and serve as a guide for the design of catalytic tests for performance optimization. From equation (1) and Extended Data Fig. 5, it is possible, for a given stirrer geometry (stirrer type and *D*), to select the stirring rate (*N*) and torque (*τ*) to be applied to deliver the desired *N*_p_ value. Nevertheless, torque control is not a widely available feature of current reactor systems for catalyst evaluation, hindering the applicability of this criterion.

2 $$\eta =\frac{{{\rm{Yield}}\;{\rm{C}}}_{1}-{{\rm{C}}}_{45}}{{({\rm{Yield}}\;{\rm{C}}_{1}-{{\rm{C}}}_{45})}_{\max }}$$

**Fig. 5:**
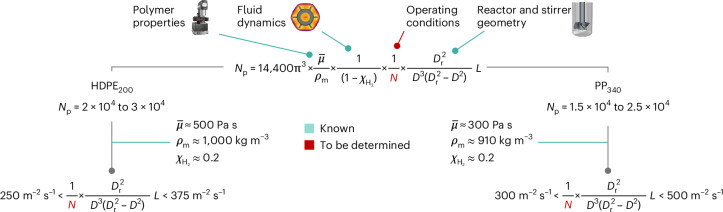
Application of the developed criteria for maximizing the effectiveness factor. Parameters that can be approximated under typical reaction conditions or are known a priori are indicated. Ranges of optimal stirring rates for a given reactor and stirrer geometry can thus be calculated. ${\bar{\mu }}$ refers to the average viscosity of the melt, *D*_r_ refers to the diameter of the stirrer and *L* to the height of the stirrer blades (Extended Data Table 1).

Use of the concentric cylinders model to describe the stirrer geometry (Supplementary Note 1) gives access to analytical relationships between viscosity, shear rate and torque, leading to an alternative expression for *N*_p_ (Fig. 5 and Supplementary Note 11) that now includes contributions from the melt properties, stirring rate, fluid dynamics (through $\chi_{{\rm{H}}_2}$), and reactor and stirrer geometry (through *D*, *D*_r_ and *L*; Extended Data Fig. 1). All of the variables are either directly observable or design parameters, except $\chi_{{\rm{H}}_2}$, which is available from Extended Data Fig. 1 and Supplementary Table 12 and depends on the stirrer type and plastic under treatment. Practitioners of catalysis can thus select appropriate combinations of stirring rate and reactor geometry to achieve the optimal *N*_p_ ranges for a certain plastic. We note that deviations from the optimal range led to differences of up to 85% in activity and 40% in selectivity (Fig. 4a).

In the most common case where the geometries of the stirrers and reactor are given, a first approximation to the optimal ranges of *N*_p_ can be obtained from the values provided in Fig. 5. Melt densities and average viscosities at typical operation temperatures (Fig. 1b) and a reasonable value for $\chi_{{\rm{H}}_2}$ of ∼0.2 (Extended Data Fig. 5) allow a straightforward calculation of stirring rate ranges. For example, in the case of *D* = 2 cm, *L* = 1 cm and *D*_r_ = 2.5 cm, the approximate ranges for high catalyst effectiveness factors would be *N* = 880–1,300 r.p.m. for HDPE_200_ and *N* = 760–1,100 r.p.m. for PP_340_ This criterion was shown to be valid in the range of most reported operation pressures (20–30 bar), with lower pressures (10 bar) showing behavior compatible with H_2_ depletion as the limiting factor (Supplementary Table 5 and Supplementary Fig. 15). These results, together with the small variation in viscosity at commonly applied temperatures (Fig. 1), make this criterion pressure- and temperature-independent under most reported conditions.

### Model scope and future directions

As the average chain length of the hydrocarbons decreases due to cleavage, so does the viscosity, spanning six orders of magnitude until reaching values close to water (Fig. 1). Thus, the ability of the criterion to predict performance as the reaction progresses was next investigated.

**Fig. 6:**
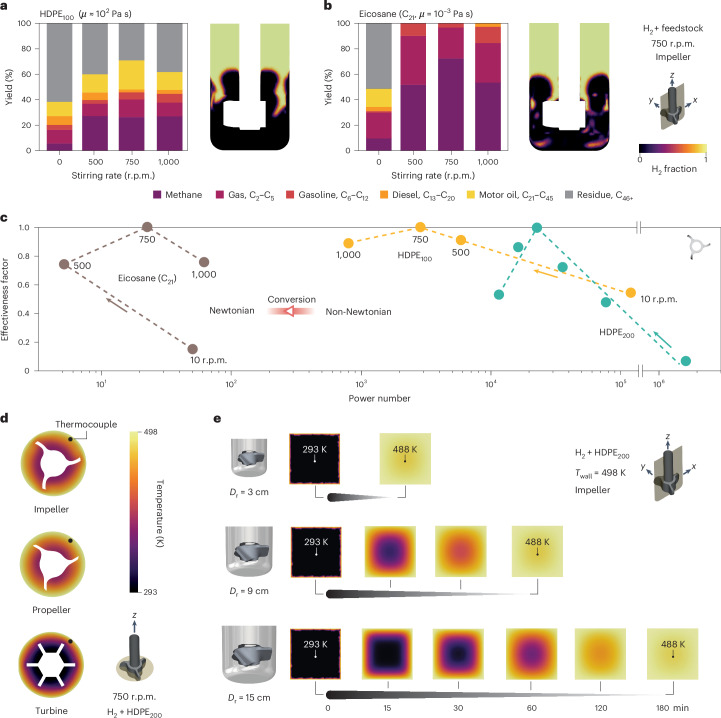
Model scope and influence of thermal gradients. **a**,**b**, Variation in the product distribution with stirring rate and two-phase CFD simulations of the hydrogen fraction in the mid *z*–*x* plane for HDPE_100_ (**a**) and eicosane (**b**). **c**, Correlation between the effectiveness factor, defined as the ratio between the yield of C_1_–C_45_ and the maximum yield of C_1_–C_45_ for HDPE and between the yield of methane and the maximum yield of methane for eicosane, and the modified power number, calculated using the simulated H_2_ fractions in Extended Data Fig. 5, using an impeller as stirrer. **d**, Temperature distribution in the mid *x*–*y* plane for different stirrer geometries when the thermocouple reaches the operation temperature (498 K, at the position indicated). **e**, Temporal evolution of the temperature distribution in the *x*–*z* plane for different reactor diameters (*D*_r_). Reaction and simulated conditions: *T* = 498 K, ${p}_{{{\rm{H}}}_{2}}$ = 20 bar and catalyst/plastic ratio = 0.05. Source data

**Extended Data Fig. 6:**
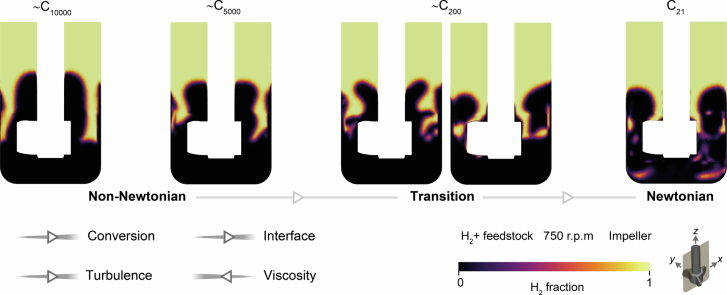
Effect of transition from Non-Newtonian to Newtonian melts in the H2-melt interface. Two-phase CFD simulations of the hydrogen fraction in the mid *z*-*x* plane for HDPE_200_ (∼C_10000_), HDPE_100_ (∼C_5000_), a generic C_200_ alkane showing a non-Newtonian character (left) and Newtonian (right), and eicosane (C_21_) resembling the evolution over the course of hydrogenolysis from Non-Newtonian to a Newtonian character. The direction of the change of other relevant features is added.

We hypothesized that the transition from the initial non-Newtonian character to a Newtonian character, facilitating the creation of turbulence^24^, may change the structure of the H_2_–melt interface. The Freely Jointed Chain model predicts a transitioning chain length of around C_200_ (Supplementary Note 5). With this in mind, we simulated stirring patterns for HDPE_100_ (non-Newtonian, a proxy for low conversion stages), a hypothetical C_200_ under Newtonian and non-Newtonian regimes, and eicosane (C_21_, Newtonian, a proxy for high conversion stages). The results clearly reflect the transition from a single H_2_–melt interface to an abundance of H_2_ bubbles populating the melt (Extended Data Fig. 6 and Supplementary Fig. 16), as supported by direct observations when turbulence starts to dominate as viscosity decreases (Supplementary Video 3). We then performed catalytic tests on HDPE_100_ and eicosane (Supplementary Table 8 and Fig. 6a,b), calculated $\chi_{{\rm{H}}_2}$ (Supplementary Table 13 and Supplementary Fig. 17) and applied the criterion (Fig. 6c). The non-Newtonian melts of HDPE_200_ and HDPE_100_ exhibited very similar trends, with identical optimal stirring rates (although different *N*_p_ due to different viscosities), whereas eicosane displayed a C-shaped relationship between effectiveness and *N*_p_, clearly suggesting the need for a different modeling strategy for the later stages of the reaction (or for the case of catalytic hydrogenolysis of surrogate molecules or the often-used very-low-molecular-weight plastics). The transition seems to occur at *N*_p_ = 10^2^–10^3^, corresponding to viscosities of around 3–30 Pa s at 1,000 r.p.m., therefore validating the proposed criterion until the later stages of the reaction.

In addition to the analysis of mass transport limitations, we also investigated heat transport constraints. We simulated the largest possible temperature gradient within the reactor during operation with three different stirrer configurations when the temperature at the thermocouple reaches the set temperature (498 K in our case, equal to that imposed on the reactor walls). Figure 6d shows the temperature distribution in the reactor, which resembles that of the Reynolds number distribution (Extended Data Fig. 2), with gradients of approximately 100 K for the best impeller and propeller geometries. This led us to conduct temporal simulations to predict the time for the gradient to reduce to less than 10 K. Figure 6e (top) shows a time of 15 min for the worst-case scenario of walls at the set temperature and the interior at room temperature at *t* = 0 with a stagnant and non-reactive melt (see Methods for more details), representing only 6% of the operation time (4 h). From a forward-looking perspective, more refined models able to stepwise predict product distributions and thus recommend optimal operation times will be possible after incorporating kinetic descriptions for the catalyst under study. Alternatively, developing operando tools to track viscosity could also guide optimal reaction times. We also highlight the generality of the applied analysis that could be adapted to future reactor architecture operating in continuous mode. In this direction, processes such as continuous reactive extrusion^53^ are first steps, which would also enable the online analysis of products.

## Discussion

The importance of stirring strategies for optimizing the potential of catalytic materials in hydrogenolysis and hydrocracking has been quantitatively established for virgin consumer-grade polyolefins. Our results show that mechanical stirring is highly recommended, even for low-molecular-weight plastics. The reaction can be assumed to develop in a millimeter-scale region next to the H_2_–melt interface for moderately and highly active catalysts. Stirring can thus be seen as a means to maximize the presence of particles in this region. Stirrer geometries largely determine the location of particles and thus performance, which decrease in the order impeller > propeller > turbine. A temperature- and pressure-independent criterion to maximize the catalyst effectiveness factor based on the observed correlation between power number and H_2_–melt interface has been developed based on the power number. For a given stirrer and reactor geometry, stirring rates for non-Newtonian melts (viscosities greater than 3–30 Pa s) can easily be calculated to operate within optimal power number values (2 × 10^4^ to 3 × 10^4^ for HDPE_200_ and 1.5 × 10^4^ to 2.5 × 10^4^ for PP_340_). Future criteria incorporating heat transport gradients will be key for the successful scale-up of this technology. This work provides readily implementable tools to maximize and tune the performance of catalysts, facilitating the standardization of catalyst evaluation and underscoring the key role of engineering considerations in catalyst development programs.

## Methods

### Rheological measurements

The viscosity of HDPE_200_ and PP_340_ (Supplementary Table 1) was measured using an Ares-G2 rheometer equipped with a separate motor and transducer (TA Instruments). A parallel plate geometry with a diameter of 25 mm was used. The temperature was regulated by convection, and flow curves were generated using a shear rate sweep from 50 s^−1^ to 0.01 s^−1^. The shear rate and viscosity were fitted assuming the plastic melts to be Carreau fluids.

### Catalyst synthesis

Commercially available titanium oxide (anatase, Sigma–Aldrich) was used as the support and calcined in ceramic crucibles in static nitrogen (5 h, 573 K and 5 K min^−1^) before synthesis. Typically, 0.30 g TiO_2_ was dry-impregnated with 1.04 cm^3^ ruthenium nitrosyl nitrate (Ru(NO)(NO_3_)_*x*_(OH)_*y*_, *x* + *y* = 3) in dilute HNO_3_ (0.015 g cm^−3^; Sigma–Aldrich) to achieve 5 wt% while mixing with a glass-coated magnetic stir bar (VWR chemicals) until the solvent had evaporated. Residual solvent was removed under vacuum (12 h, 353 K and 80 mbar). Finally, the sample was heated in nitrogen at 573 K. The prepared catalyst was then pressed and sieved into different sieve fractions (0.0−0.2, 0.2−0.4 and 0.4−0.6 mm).

### Catalyst characterization

High-angle annular dark-field scanning transmission electron microscopy and energy-dispersive X-ray spectroscopy were carried out on a probe-corrected Titan Themis microscope operated at 300 kV. Samples were pretreated to remove adventitious compounds in Ar/O_2_ plasma before being inserted into the microscope. X-ray photoelectron spectroscopy (XPS) was conducted using a Physical Electronics Quantera SXM spectrometer. Monochromatic Al Kα radiation (1,486.6 eV) generated by an electron beam (15 kV and 49.3 W) was used to irradiate the samples with a spot size of 200 μm. The finely ground samples were pressed into indium foil (99.9%, Alfa Aesar) and then mounted onto the sample holder. During measurement, electron and ion neutralizers were operated simultaneously to suppress undesired sample charging. High-resolution spectra were obtained using pass energies of 55 eV, while the Au *f*_7/2_ signal at 84 ± 0.1 eV was used for calibration. All calculations were performed with the CasaXPS (Casa software), and the relative sensitivity factors used for quantification were taken from the instrument. All spectra were deconvoluted into Gaussian–Lorentzian components after application of the Shirley background. X-ray diffraction was conducted using a Rigaku SmartLab diffractometer equipped with a D/teX Ultra 250 detector and Cu Kα radiation (*λ* = 0.1541 nm) operating in Bragg−Brentano geometry. Data were acquired in the 2*θ* range of 20–80° with an angular step size of 0.025° and a counting time of 1.5 s per step. Temperature-programmed desorption experiments were conducted using a Micromeritics AutoChem II 2920 instrument. Signals were acquired with a pre-equipped thermal conductivity detector (TCD) and an attached Pfeiffer Vacuum OmniStar GSD 320 O mass spectrometer (MS). Samples of ~0.1 g were dried at 473 K for 1 h, followed by saturation with either *n*-C_7_H_16_ or 2,4-dimethylpentane (contained in an attached vaporizer). In the subsequent desorption experiment, a heating ramp (313–873 K at 10 K min^−1^) was applied while using both the TCD and MS to monitor the evolution of the probe gas.

### Catalyst evaluation

The catalysts were evaluated in a parallel batch reactor set-up (BüchiGlasUster) fitted with an electrical heating jacket and active cooling unit (chilled water system). Typically, 0.5 g virgin polyethylene or polypropylene and 0.025 g catalyst were placed inside a glass inset, which was then placed inside the reactor (Supplementary Fig. 1). Catalyst particles were always found at the bottom of the reactor after reaction regardless of the catalyst activity. The distance between the base of the stirrer and the bottom of the reactor was minimized, and it is highly recommended to use a volume of melt that closely matches the envelope of the stirrer to minimize melt zones between the stirrer blades and reactor walls, where stirring is highly ineffective due to the shear-thinning nature of molten plastics. The reactors were flushed first with nitrogen and then with hydrogen before pressurization to the desired pressure. All reactor systems were equipped with mechanical stirring, temperature/pressure control systems and gas sampling lines. A schematic of the reactor set-up, including the most relevant dimensions, is shown in Extended Data Fig. 1, and the experimental limitations are described in Supplementary Note 2. Instantaneous values of temperature, pressure and stirring torque were recorded using the SYSTAG Flexsys software^54^. The temperature in the reactor was measured using a thermocouple placed in the space (immersed in the polyolefin melt) between the stirrer and reactor wall. Before the reaction, the reactor was weighed along with all its content. The reaction mixture was heated and stirred (at 498 K and 750 r.p.m. unless otherwise specified) for a set reaction time. After the reaction, the vessels were cooled with circulating chilled water. Three different commercially available stirrer types (acquired from BüchiGlasUster) were used, namely, a turbine, propeller and impeller. They all had the same blade height (1 cm) and diameter (2.8 cm). The glass inset containing the reaction mixture had a diameter of 3 cm (Extended Data Fig. 1) and was inserted into a stainless-steel reactor placed in a heating jacket. The glass inset was fabricated so that its outer diameter closely fitted the inner diameter of the reactor (Supplementary Fig. 1). A detailed description of the experimentally available range of operating conditions is provided in Supplementary Note 2.

### Product analysis

The gaseous products were collected from the headspace of the reactor using a sampling cylinder and analyzed using a gas chromatograph (HP Agilent 6890) equipped with a 25 m × 0.53 mm × 20 μm column (Agilent J&W PoraPlot Q column) and flame ionizing detector (FID). A temperature ramp of 308–573 K (5 K min^−1^) was applied, while the inlet and FID were held at 573 K and 473 K, respectively. The gas chromatograph (GC) columns were calibrated as per a procedure reported elsewhere for products C_1_–C_45_ (ref. ^55^); the calibration was performed for products C_1_–C_5_, detected by FID, using a standard refinery gas mixture (Agilent P/N 5080-8755). The reactor inset was then weighed to calculate the amount of gas formed. The products remaining inside the inset were dissolved in dichloromethane using sonication and filtered using a syringe for GC-FID and ^1^H NMR analysis. GC-FID analysis was performed on a GC (HP Agilent 6890) equipped with a 15 m × 0.25 mm × 0.10 μm column (HP DB-5 HT). A temperature ramp of 313–648 K (4 K min^−1^) was applied, while the FID detector was held at 613 K. The initial and final hold times were set at 2 and 10 min, respectively. Calibration was performed for C_7_–C_40_ alkanes using a certified reference mixture (C_7_–C_40_ in hexane, 1 mg cm^−3^, traceCERT, Sigma–Aldrich). For each ^1^H NMR experiment, conducted on a 300 MHz Bruker Ultrashield spectrometer, 0.45 cm^3^ of sample and 0.05 cm^3^ of [D_2_]dichloromethane were mixed and analyzed using a solvent suppression method reported elsewhere^2^. The signals in the aliphatic region of the ^1^H NMR spectra were numerically integrated to identify the ratio of primary to secondary carbon atoms. The areas corresponding to each carbon type were normalized by the number of hydrogen atoms bonded to the carbon.

### CFD simulations

CFD simulations were performed with Ansys Academic Research Fluent (Release 2023 R1) using a double-precision steady-state solver. Supplementary Note 3 describes the scope and limitations of the simulations when applied to polyolefin hydrogenolysis. The geometry of the reactor was created in Ansys SpaceClaim on a 1:1 scale with the actual dimensions. The geometry was meshed using the watertight geometry workflow of Ansys Fluent Meshing, resulting in 0.5 million polyhedral cells. The stirring within the reactor was simulated using the moving reference frame method. Fluid flow was computed using the renormalized group k-ε model with swirl-dominated flow and Menter–Lechner near-wall treatment. The experimentally measured viscosity of the plastic melt was modeled using the Carreau–Yasuda model and implemented in Fluent as an interpreted function. The power number was calculated as a user-defined function within Fluent. The pressure–velocity coupled solver used the Rhie–Chow momentum-based flux type auto-selected by Fluent, which was run until residual values of 10^−5^ were reached for continuity, *x*, *y* and *z* velocities, turbulent kinetic energy (*k*) and the rate of dissipation of kinetic energy (*e*).

The two-phase system of hydrogen and molten polyolefin was simulated using the volume-of-fluid (VOF) method with sharp interfaces, continuum surface stress and the no-slip condition. The VOF simulations were achieved by solving the continuity equation (3), where *ρ*_*q*_, *α*_*q*_ and **v**_*q*_ refer to the density, volume fraction and velocity of a given phase *q*, ${\dot{m}}_{sq}$ and ${\dot{m}}_{qs}$ refer to the mass transfer between phases *q* and *s* within the multiphase system, *t* refers to time and *S* indicates a mass transfer source (none in this case). The validity of this equation is subject to equation (4). A condition of no mass transfer between the phases was assumed, which results in equation (5). The volume fraction of the secondary phase (in this case molten plastics) was first computed, followed by the calculation of the primary phase (hydrogen) using the constraint that the sum of the volume fractions of all phases must be 1. The volume fraction equation was solved in this case using an implicit scheme for time discretization that uses equation (6), where *V* refers to the volume of the cell, *U*_*f*_ refers to the volume flux through the face, based on a normal velocity, *n* refers to the previous iteration step, while *n* + 1 is the current iteration step, *α*_*q,f*_ is the face value of the *q*th volume fraction computed through either the first- or second-order upwind scheme and Δ*t* is the infinitesimally small time step considered for iterative computation. The volume used to compute the H_2_ fraction was a cylinder delimited by the free surface of the melt, the reactor walls and reached 2 mm below the stirrer base after preliminary tests to determine the volume showing the best compromise to account for the H_2_–melt interface under all of the tested conditions.

3 $$\frac{1}{{\rho }_{q}}\left[\frac{\partial }{\partial t}({\alpha }_{q}{\rho }_{q})+\nabla ({\alpha }_{q}{\rho }_{q}{\bf{v}}_{q})=S{\alpha }_{q}+\mathop{\sum }\limits_{s=1}^{n}({\dot{m}}_{sq}-{\dot{m}}_{qs})\right]$$

4 $$\mathop{\sum }\limits_{q=1}^{n}{\alpha }_{q}=1$$

5 $$\frac{\partial }{\partial t}({\alpha }_{q}{\rho }_{q})+\nabla ({\alpha }_{q}{\rho }_{q}{\bf{v}}_{q})=0$$

6 $$\frac{{\alpha }_{q}^{n+1}{\rho }_{q}^{n+1}-{\alpha }_{q}^{n}{\rho }_{q}^{n}}{\Delta t}V+\sum _{f}{\rho }_{q}^{n+1}{U}_{\!f}^{\;n+1}{\alpha }_{q,\,f}^{n+1}=\left[S{\alpha }_{q}+\mathop{\sum }\limits_{s=1}^{n}({\dot{m}}_{sq}-{\dot{m}}_{qs})\right]V$$

Particles within the melt were simulated using the discrete phase modeling method of Ansys Fluent with the particles being allowed to interact with the continuous phase. Particles were introduced at the bottom of the vessel during simulations to reflect the fact that they were always found at the bottom of the reactor after reaction regardless of the catalyst activity. Virtual mass force and pressure gradient force models were employed along with two-way turbulence coupling. The trajectory of each particle was computed using the force balance on the particle provided by equation (7), where *v*_p_ and *v*_m_ are the velocities of the particles and fluid (multiphase mix of plastic melt and hydrogen), respectively, *g*_*x*_ is the gravitational acceleration (9.81 m s^−2^), and *ρ*_p_ and *ρ*_m_ are the densities of the particle and fluid, respectively. The drag force (*F*_D_) was computed using equation (8), where *C*_D_ is the coefficient of drag, Re_p_ is the particle Reynolds number (computed with equation (9)) and *d*_p_ is the particle diameter. *F*_*x*1_ represents the virtual mass force (equation (10)) and *F*_*x*2_ is the pressure gradient force (equation (11)).

7 $$\frac{{\rm{d}}{v}_{{\rm{p}}}}{{\rm{d}}t}={F}_{{\rm{D}}}({v}_{{\rm{m}}}-{v}_{{\rm{p}}})+\frac{{g}_{x}(\;{\rho }_{{\rm{p}}}-{\rho }_{{\rm{m}}})}{{\rho }_{{\rm{m}}}}+{F}_{\!x1}+{F}_{\!x2}$$

8 $${F}_{{\rm{D}}}=\frac{18\mu {C}_{{\rm{D}}}{\mathrm{Re}}_{{\rm{p}}}}{24{\rho }_{{\rm{p}}}{d}_{{\rm{p}}}^{\,2}}$$

9 $${{\rm{Re}}}_{{\bf{p}}}=\frac{{\rho }_{{\rm{m}}}{d}_{{\rm{p}}}({v}_{{\rm{p}}}-{v}_{{\rm{m}}})}{\mu }$$

10 $${F}_{x1}=\frac{1}{2}\frac{{\rho }_{{\rm{m}}}}{{\rho }_{{\rm{p}}}}\frac{{\rm{d}}}{{\rm{d}}t}({v}_{{\rm{m}}}-{v}_{{\rm{p}}})$$

11 $${F}_{x2}=\frac{1}{2}\left(\frac{{\rho }_{{\rm{m}}}}{{\rho }_{{\rm{p}}}}\right){v}_{{{\rm{p}}}_{i}}\frac{\partial v}{\partial {x}_{i}}$$

The sources of the discrete phase model were updated every flow iteration. The particles were injected into the two-phase system of molten polyolefin and hydrogen after the VOF model had been fully resolved. Particle injection was achieved using an injection file generated by a Python script to randomly generate particles at coordinates (*x*, *y*, *z*) near the base of the stirrer.

Hydrogen diffusion across a static polyolefin melt was simulated with the COMSOL Multiphysics software using a time-dependent dilute species diffusion model (in all three dimensions) constructed using Fick’s law of diffusion. The concentration of hydrogen was varied from 25 to 100 mol m^−3^, corresponding to different pressures of hydrogen. The diffusivity of hydrogen through the polyolefin was varied between 10^−8^ and 5 × 10^−8^ m^2^ s^−1^, corresponding to different polyolefin *M*_w_, as explained in Supplementary Note 6. The time-dependent temperature profile in a static polyolefin melt (with the reactor wall temperature set at 498 K) was simulated with the COMSOL Multiphysics software^56^.

The steady-state temperature profiles in polyolefin melts stirred with various stirrer geometries were simulated by adding energy dissipation equations to the volume-of-fluid and discrete particle method (VOF-DPM) model in ANSYS Fluent (ref. ^57^). The heat transfer coefficients and heat capacities were taken from libraries embedded within ANSYS. As the stirring simulations were always conducted under steady-state conditions, the reported results correspond to the case where the temperature at the position of the thermocouple reached the set temperature.

## Supplementary information

Supplementary Information Supplementary Notes 1–12, Figs. 1–17, Tables 1–13 and references.

Supplementary Video 1 Mixing with a magnetic stirrer: water, LDPE_35_ and HDPE_100_.

Supplementary Video 2 Mixing with a magnetic stirrer: water, PP_12_ and PP_340_.

Supplementary Video 3 Mixing with a mechanical stirrer: eicosane, LDPE_35_ and HDPE_100_.

Supplementary Video 4 Trajectories of 0.2 mm particles at 1,750 r.p.m. for impeller, propeller and turbine stirrers.

## Source data

Source Data Fig. 1 Raw data for plots in Fig. 1.

Source Data Fig. 2 Raw data for plots in Fig. 2.

Source Data Fig. 3 Raw data for plots in Fig. 3.

Source Data Fig. 4 Raw data for plots in Fig. 4.

Source Data Fig. 6 Raw data for plots in Fig. 6.

Source Data Extended Data Fig. 2 Raw data for plots in Extended Data Fig. 2.

Source Data Extended Data Fig. 3 Raw data for plots in Extended Data Fig. 3.

Source Data Extended Data Fig. 4 Raw data for plots in Extended Data Fig. 4.

Source Data Extended Data Fig. 5 Raw data for plots in Extended Data Fig. 5.

## Data Availability

The data presented in the figures of this paper are publicly available via Zenodo at 10.5281/zenodo.10812922 (ref. ^58^). Other supporting data are available from the corresponding authors upon request. Source data are provided with this paper.

## Extended data

**Extended Data Table 1:** Definitions and ranges (I) of variables used in this study

	Symbol	Meaning	Formula	Units	Typical values
Polyolefin properties	*μ*	Local viscosity of polymer melt	-	Pa s	200-6,000 (PP_340_) 400-6,500 (HDPE_200_)
	$\bar{\mu }$	Average viscosity of polymer melt under typical stirring rates	-	Pa s	300 (PP_340_) 500 (HDPE_200_)
	*D*_H2_	H_2_ diffusivity in molten polymer		m^2^ s^−1^	∼10^−8^
	*ρ*_m_	Density of polymer melt	-	kg m^−3^	910 (PP_340_) 1,000 (HDPE_200_)
Testing conditions	*T*	Temperature	-	K	473-523
	*p*_H2_	Pressure of hydrogen	-	bar	10-50
	*N*	Stirring rate	-	rpm	0-2500
	*ω*	Rotation rate	$\omega =\frac{2\pi N}{60}$	rad s^−1^	0-260
	*τ*	Torque	-	N m	0.06-0.1
	*γ*	Shear rate	$\gamma =\frac{2\tau }{\pi L{D}^{2}\bar{\mu }}\approx \frac{N{D}_{r}^{2}}{60\pi ({D}_{r}^{2}-{D}^{2})}$	s^−1^	0-2,000 for lab mechanical stirrers
Geometry	*d*_p_	Catalyst particle diameter		m	<10^−3^
	*D*	Stirrer diameter	-	m	0.01-0.05 for lab reactors
	*L*	Height of stirrer blades	-	m	0.01-0.05 for lab reactors
	*D*_r_	Inner reactor diameter	-	m	0.01-0.10 for lab reactors
Chemical reaction	*r*	Volumetric reaction rate	*r* = *k*_r_*c*_H2_	mol_H2_·s^−1^·m^−3^	∼0.1-10 in this study
	*c*_H2_	H_2_ concentration at the interface	-	mol m^−3^	∼50 in this study (Henry’s law)
	*k*_r_	Rate coefficient	-	s^−1^	∼0.003-0.15 in this study
	*λ*	Characteristic length of reaction front	$\lambda =\sqrt{\frac{{D}_{{\rm{H2}}}}{{k}_{{\rm{r}}}}}$	m	10^−4^ -10^−3^
	*η*	Effectiveness factor	$\eta =\frac{{{\rm{YieldC}}}_{1}{-{\rm{C}}}_{45}}{{{({\rm{YieldC}}}_{1}{-{\rm{C}}}_{45})}_{\max }}$	-	0-1

Variables used in this study to define polyolefin properties, catalytic testing conditions, stirrer and vessel geometry, and chemical reaction.

**Extended Data Table 2:** Definitions and ranges (II) of variables used in this study

Symbol	Meaning	Formula	Units	Typical values
***v***_**p**,***z***_	Catalyst particle velocity along the *z* axis	-	m s^−1^	0.0-0.2
***v***_**tip**_	Velocity of the tip of the stirrer blade	${v}_{{\rm{tip}}}=\frac{\pi ND}{60}$	m s^−1^	0-5
**Ar**	Archimedes number	${\rm{Ar}}=\frac{{\rm{g}}{{d}_{{\rm{p}}}}^{3}{\rho }_{{\rm{m}}}({\rho }_{{\rm{p}}}-{\rho }_{{\rm{m}}})}{{\mu }^{2}}$	-	10^−7^ -10^−8^
**Stk**	Stokes number	${\rm{Stk}}=\frac{{\rho }_{{\rm{m}}}{{d}_{{\rm{p}}}}^{2}{v}_{{\rm{tip}}}}{18\mu \lambda }$	-	10^−2^ -10^0^
**Ha**	Hatta number	${\rm{Ha}}=\frac{\sqrt{{k}_{{\rm{r}}}{D}_{{{\rm{H}}}_{2}}}}{{k}_{{{\rm{H}}}_{2}}}$	-	1-7
***K***_**s**_	Shape factor	${K}_{s}=\frac{{v}_{p,z,\max }}{{v}_{tip}}$	-	0.001-0.1 (dependent on stirrer type)
**Re**_**p**,***z*****,max**_	Maximum Reynolds number of catalyst particles along the *z* axis	$$\begin{array}{c}{\mathrm{Re}}_{p,z,\max }=\frac{{\rho }_{{\rm{m}}}{v}_{p,z,\max }{d}_{p}}{\mu }\\ \approx \frac{\pi {\rho }_{m}}{60\bar{\mu }}N{K}_{{\rm{s}}}D{d}_{p}\end{array}$$	-	0-1.5 × 10^−3^ for tested configurations
***χ***_**H2**_	Volumetric H_2_ fraction in polymer melt	-	-	0-0.3 for tested configurations
**N**_**p**_	Power number	$$\begin{array}{c}{{\rm{N}}}_{{\rm{p}}}=\frac{\tau \omega }{\bar{\rho }{N}^{3}{D}^{5}}=\frac{7200\pi \tau }{\bar{\rho }{N}^{2}{D}^{5}}\\ \approx \frac{14400{\pi }^{3}\bar{\mu }{D}_{{\rm{r}}}^{2}L}{(1-{\chi }_{{\rm{H2}}}){\rho }_{{\rm{m}}}{D}^{3}({D}_{{\rm{r}}}^{2}-{D}^{2})N}\end{array}$$	-	0-∞

Variables used in this study to define catalyst particles motion and fluid dynamics of the melt.

## Code availability

No new code was generated in this work.

## References

[1] Hayden, B., Canuel, N. & Shanse, J. What was brewing in the Natufian? An archaeological assessment of brewing technology in the Epipaleolithic. *J. Archaeol. Method Theory* **20**, 102–150 (2013).

[2] Williams, F. J. Widnes and the early chemical industry 1847–71. A case of occupational mobility in the industrial revolution. *Trans. Hist. Soc. Lancs. Ches.* **134**, 89–105 (1984).

[3] Damköhler, G. Einflüsse der Strömung, Diffusion und des Wärmeüberganges auf die Leistung von Reaktionsöfen: I. Allgemeine Gesichtspunkte für die Übertragung eines chemischen Prozesses aus dem Kleinen ins Große. *Z. Elektrochem. Angew. Phys. Chem.* **42**, 846–862 (1936).

[4] Thiele, E. W. Relation between catalytic activity and size of particle. *Ind. Eng. Chem. Res.* **31**, 916–921 (1939).

[5] Pereira, C. J., Carberry, J. J. & Varma, A. Uniqueness criteria for first order catalytic reactions with external transport limitations. *Chem. Eng. Sci.* **34**, 249–255 (1979).

[6] Carberry, J. J. in *Chemical and Catalytic Reaction Engineering* 474–519 (Dover Publications, 1991).

[7] Geyer, R., Jambeck, J. R. & Law, K. L. Production, use, and fate of all plastics ever made. *Sci. Adv.* **3**, e1700782 (2017). 28776036 10.1126/sciadv.1700782 PMC5517107

[8] Mitchell, S., Martín, A. J. & Pérez-Ramírez, J. Transcending scales in catalysis for sustainable development. *Nat. Chem. Eng.* **1**, 13–15 (2024).

[9] Shirazimoghaddam, S., Amin, I., Faria Albanese, J. A. & Shiju, N. R. Chemical recycling of used PET by glycolysis using niobia-based catalysts. *ACS Eng. Au* **3**, 37–44 (2023). 36820227 10.1021/acsengineeringau.2c00029 PMC9936547

[10] Sullivan, K. P. et al. Mixed plastics waste valorization through tandem chemical oxidation and biological funneling. *Science* **378**, 207–211 (2022). 36227984 10.1126/science.abo4626

[11] Martín, A. J., Mondelli, C., Jaydev, S. D. & Pérez-Ramírez, J. Catalytic processing of plastic waste on the rise. *Chem* **7**, 1487–1533 (2021).

[12] Vollmer, I. et al. Beyond mechanical recycling: giving new life to plastic waste. *Angew. Chem. Int. Ed.* **59**, 15402–15423 (2020). 10.1002/anie.201915651 PMC7497176 32160372

[13] Li, H. et al. Expanding plastics recycling technologies: chemical aspects, technology status and challenges. *Green Chem.* **24**, 8899–9002 (2022).

[14] Hancock, J. N. & Rorrer, J. E. Hydrogen-free catalytic depolymerization of waste polyolefins at mild temperatures. *Appl. Catal. B* **338**, 123071 (2023).

[15] Jaydev, S. D., Usteri, M. E., Martín, A. J. & Pérez-Ramírez, J. Identifying selective catalysts in polypropylene hydrogenolysis by decoupling scission pathways. *Chem Catal.* **3**, 100564 (2023).

[16] Jaydev, S. D., Martín, A. J. & Pérez-Ramírez, J. Direct conversion of polypropylene into liquid hydrocarbons on carbon-supported platinum catalysts. *ChemSusChem* **14**, 5179–5185 (2021). 34553832 10.1002/cssc.202101999

[17] Kots, P. A. et al. Polypropylene plastic waste conversion to lubricants over Ru/TiO_2_ catalysts. *ACS Catal.* **11**, 8104–8115 (2021).

[18] Celik, G. et al. Upcycling single-use polyethylene into high-quality liquid products. *ACS Cent. Sci.* **5**, 1795–1803 (2019). 31807681 10.1021/acscentsci.9b00722 PMC6891864

[19] Abbas-Abadi, M. S., Haghighi, M. N. & Yeganeh, H. Evaluation of pyrolysis product of virgin high density polyethylene degradation using different process parameters in a stirred reactor. *Fuel Process. Technol.* **109**, 90–95 (2013).

[20] Elordi, G., Olazar, M., Lopez, G., Artetxe, M. & Bilbao, J. Product yields and compositions in the continuous pyrolysis of high-density polyethylene in a conical spouted bed reactor. *Ind. Eng. Chem. Res.* **50**, 6650–6659 (2011).

[21] Conesa, J. A., Font, R., Marcilla, A. & Garcia, A. N. Pyrolysis of polyethylene in a fluidized bed reactor. *Energy Fuels* **8**, 1238–1246 (1994).

[22] Mastral, J. F., Berrueco, C. & Ceamanos, J. Pyrolysis of high-density polyethylene in free-fall reactors in series. *Energy Fuels* **20**, 1365–1371 (2006).

[23] Todd, D. B. in *Handbook of Industrial Mixing* 987–1025 (John Wiley, 2003).

[24] Zlokarnik, M. in *Stirring* 76–96 (Wiley-VCH, 2001).

[25] Ge, J. & Peters, B. Mass transfer in catalytic depolymerization: external effectiveness factors and serendipitous processivity in stagnant and stirred melts. *Chem. Eng. J.* **466**, 143251 (2023).

[26] Rejman, S. et al. Transport limitations in polyolefin cracking at the single catalyst particle level. *Chem. Sci.* **14**, 10068–10080 (2023). 37772101 10.1039/d3sc03229a PMC10529962

[27] Liu, K. & Meuzelaar, H. L. C. Catalytic reactions in waste plastics, HDPE and coal studied by high-pressure thermogravimetry with on-line GC/MS. *Fuel Process. Technol.* **49**, 1–15 (1996).

[28] Serrano, D. P., Aguado, J., Escola, J. M. & Rodríguez, J. M. Influence of nanocrystalline HZSM-5 external surface on the catalytic cracking of polyolefins. *J. Anal. Appl. Pyrolysis* **74**, 353–360 (2005).

[29] Jerdy, A. C. et al. Deconvoluting the roles of polyolefin branching and unsaturation on depolymerization reactions over acid catalysts. *Appl. Catal. B* **337**, 122986 (2023).

[30] Tennakoon, A. et al. Catalytic upcycling of high-density polyethylene via a processive mechanism. *Nat. Catal.* **3**, 893–901 (2020).

[31] Lee, Y. H., Sun, J., Scott, S. L. & Abu-Omar, M. M. Quantitative analyses of products and rates in polyethylene depolymerization and upcycling. *STAR Protoc.* **4**, 102575 (2023). 37729056 10.1016/j.xpro.2023.102575 PMC10517283

[32] Henzler, H.-J. & Obernosterer, G. Effect of mixing behaviour on gas-liquid mass transfer in highly viscous, stirred non-Newtonian liquids. *Chem. Eng. Technol.* **14**, 1–10 (1991).

[33] Kang, Q., Liu, J., Feng, X., Yang, C. & Wang, J. Isolated mixing regions and mixing enhancement in a high-viscosity laminar stirred tank. *Chin. J. Chem. Eng.* **41**, 176–192 (2022).

[34] Bremner, T., Rudin, A. & Cook, D. G. Melt flow index values and molecular weight distributions of commercial thermoplastics. *J. Appl. Polym. Sci.* **41**, 1617–1627 (1990).

[35] Ng, K. Y. & Erwin, L. Experiments in extensive mixing in laminar flow. I. Simple illustrations. *Polym. Eng. Sci.* **21**, 212–217 (1981).

[36] Cortada-Garcia, M., Dore, V., Mazzei, L. & Angeli, P. Experimental and CFD studies of power consumption in the agitation of highly viscous shear thinning fluids. *Chem. Eng. Res. Des.* **119**, 171–182 (2017).

[37] Magnetic stirrers frequently asked questions. *Fisher Scientific* https://www.fishersci.se/se/en/scientific-products/featured-categories/magnetic-stirrers/frequently-asked-questions.html (2024).

[38] Viscous slurry machinery. *Siehe* https://www.sieheindustry.com/product_category/viscous-slurry-machinery (2024).

[39] Jaydev, S. D. et al. Consumer grade polyethylene recycling via hydrogenolysis on ultrafine supported ruthenium nanoparticles. *Angew. Chem. Int. Ed.* **63**, e202317526 (2023). 10.1002/anie.202317526 38105396

[40] Chen, L. et al. Effect of reaction conditions on the hydrogenolysis of polypropylene and polyethylene into gas and liquid alkanes. *React. Chem. Eng.* **7**, 844–854 (2022).

[41] Chen, L., Moreira, J. B., Meyer, L. C. & Szanyi, J. Efficient and selective dual-pathway polyolefin hydro-conversion over unexpectedly bifunctional M/TiO_2_-anatase catalysts. *Appl. Catal. B* **335**, 122897 (2023).

[42] Rorrer, J. E., Beckham, G. T. & Román-Leshkov, Y. Conversion of polyolefin waste to liquid alkanes with Ru-based catalysts under mild conditions. *JACS Au* **1**, 8–12 (2021). 34467267 10.1021/jacsau.0c00041 PMC8395642

[43] Rorrer, J. E., Troyano-Valls, C., Beckham, G. T. & Román-Leshkov, Y. Hydrogenolysis of polypropylene and mixed polyolefin plastic waste over Ru/C to produce liquid alkanes. *ACS Sustain. Chem. Eng.* **9**, 11661–11666 (2021).

[44] Shinohara, K., Yanagisawa, M. & Makida, Y. Direct observation of long-chain branches in a low-density polyethylene. *Sci. Rep.* **9**, 9791 (2019). 31278359 10.1038/s41598-019-46035-9 PMC6611765

[45] Gedde, U. W. et al. Molecular structure, crystallization behavior, and morphology of fractions obtained from an extrusion grade high-density polyethylene. *Polym. Eng. Sci.* **28**, 1289–1303 (1988).

[46] Sakai, T. in *Physics of Polymer Gels* 1–22 (Wiley‐VCH, 2020).

[47] Keller, A. & O’Connor, A. A study on the relation between chain folding and chain length in polyethylene. *Polymer* **1**, 163–168 (1960).

[48] Levenspiel, O. in *Chemical Reaction Engineering* 566–606 (John Wiley, 1999).

[49] Brennen, C. E. in *Fundamentals of Multiphase Flow* 252–271 (Cambridge Univ. Press, 2005).

[50] Mason, A. H. et al. Rapid atom-efficient polyolefin plastics hydrogenolysis mediated by a well-defined single-site electrophilic/cationic organo-zirconium catalyst. *Nat. Commun.* **13**, 7187 (2022). 36418305 10.1038/s41467-022-34707-6 PMC9684440

[51] Edenfield, W. C. et al. Rapid polyolefin plastic hydrogenolysis mediated by single-site heterogeneous electrophilic/cationic organo-group IV catalysts. *ACS Catal.* **14**, 554–565 (2024).

[52] Hemrajani, R. R. & Tatterson, G. B. in *Handbook of Industrial Mixing* 345–390 (John Wiley, 2003).

[53] Chandrasekaran, S. et al. Recent advances in metal sulfides: from controlled fabrication to electrocatalytic, photocatalytic and photoelectrochemical water splitting and beyond. *Chem. Soc. Rev.* **48**, 4178–4280 (2019). 31206105 10.1039/c8cs00664d

[54] SYSTAG. System Technik AG https://www.systag.ch/de/labor-reaktor-systeme/produkte/flexyconcept/flexysys/ (accessed 14 August 2024).

[55] Rome, K. & Mcintyre, A. Intelligent use of relative response factors in gas chromatography-flame ionisation detection. *Chromatogr. Today* 52–56 (2012).

[56] Theory for heat transfer in fluids. *COMSOL* https://doc.comsol.com/6.1/docserver/#!/com.comsol.help.heat/heat_ug_theory.07.008.html (2024).

[57] Energy equation. *ANSYS* https://www.afs.enea.it/project/neptunius/docs/fluent/html/th/node302.htm (2024).

[58] Jaydev, S. D., Martín, A. J., García, D., Chikri, K. & Pérez-Ramírez, J. Assessment of transport phenomena in catalyst effectiveness for chemical polyolefin recycling. *Zenodo*10.5281/zenodo.10812922 (2024). 10.1038/s44286-024-00108-3 PMC11420077 39323546

