# Supplementary material for: Assessment of transport phenomena in catalyst effectiveness for chemical polyolefin recycling
Source: Nat Chem Eng. 2024 Aug 28;1(9):565–75. doi: 10.1038/s44286-024-00108-3 (PMC11420077; doi:10.1038/s44286-024-00108-3)
Supplement: Supplementary file 1 — Supplementary Notes 1–12, Figs. 1–17, Tables 1–13 and references. [file 44286_2024_108_MOESM1_ESM.pdf]

nature chemical engineering

Supplementary information https://doi.org/10.1038/s44286-024-00108-3

Assessment of transport phenomena in catalyst effectiveness for chemical polyolefin recycling

In the format provided by the authors and unedited

Table of contents

Supplementary Notes……………………………………………………………………………...2
Supplementary Figures..………………………………………………………………….......16
Supplementary Tables ………………………………………………………………………33
Supplementary References ……………………………………………………………………47

Supplementary Notes

Supplementary Note 1 | Relationship between viscosity, shear rate, and torque.

Viscosity (μ), shear stress (λ) and shear rate (γ) can be locally linked for a non-Newtonian fluid by the Equation S1.

$$\mu = \frac{\lambda}{\gamma} \tag{S1}$$

In fluid mixing processes, such as those in stirred vessels, the shear rate is influenced by various factors, including velocity gradients, impeller design, flow characteristics, and fluid properties. Viscosity gradients arise due to the variation in fluid velocities within the mixing system. Near the impeller blades, these gradients can be significant, leading to higher shear rates. Herein, we considered the simple model of a rotating cylinder[1] with D and L as the diameter and height L, respectively, in a concentric cylindrical vessel of diameter D_r. This model enabled an analytical relationship between applied torque (τ) and between shear stress (Equation S2) and shear rate and stirrer and vessel geometry (Equation S3), that were applied to the analysis of the three types of stirrers studied in this work,

$$\lambda = \frac{2\tau}{\pi L D^2} \tag{S2}$$

$$\gamma = \frac{\pi N D^2 D_r^2}{60 x^2 (D_r^2 - D^2)} \tag{S3}$$

where x represents the radial distance from the vertical rotation axis. For the case of small clearance between the tip of the blade and the vessel wall, the simplification $x = D/2$ and rearranging of Equations S1-S3 leads to a simplified expression for the representative value of viscosity in terms of operating conditions and geometry (Equation S4), which allows estimation of viscosity when torque is measured during catalyst testing.

$$\mu = \frac{\tau (D_r^2 - D^2)}{30 \pi^2 N D^2 D_r^2 L} \tag{S4}$$

This set of relationships was used in **Fig. 1c** to estimate stirring rates in our setup from shear rates obtained from rheological analysis, revealing that under typical stirring rates, viscosity can be considered independent from stirring rate and temperature.

Supplementary Note 2 | Experimentally available range of operating conditions.

The parallel reactor setup used in this study comprised three reactors that can be operated independently as shown in **Supplementary Fig. 1**. The range of operating conditions is representative of the current state of the art and is provided below for reference when interpreting CFD simulations.

The maximum amount of processable plastic was about 1 g. Larger amounts led to poor conversion due to inefficient overall stirring originated from the Non-Newtonian nature of the polymer melt, showing much larger viscosities (and thus decreased Reynolds numbers) in regions separated from the blades, where the shear rate is lower. The maximum operating temperature is 623 K mainly due to limitations of the o-ring sealings of the vessel. The maximum feasible hydrogen pressure is 150 bar, which suggests the lack of operational limitations on this aspect. The stirring capabilities are limited to the maximum torque provided by the engine (70 N cm), that resulted in a maximum stirring rate of ca. 1500 rpm under typical conditions for consumer grade plastics like $HDPE_{200}$ and PP_{340}. Monitoring of the torque is possible in our setup, enabling indirect calculation of the average viscosity of the melt over the course of the experiment, as explained in the **Supplementary Note 1**. The flow regime in all cases was laminar, since a minimum stirring rate of 10^5 s^{-1} (~10^6 rpm) is required to achieve an average Reynolds number of 10^4 giving access to turbulent flow[2] (Equation S5):

$$\mathrm{Re} = \frac{\rho_\mathrm{m} v D}{\mu} = \frac{\rho_\mathrm{m} N D^2}{\mu} \approx \frac{10^3 N \left(10^{-1}\right)^2}{10^2} = 10^{-1} N \geq 10^4 \Rightarrow N \geq 10^5\ \mathrm{s}^{-1} \qquad \text{(S5)}$$

where the velocity of the stirrer tip has been taken as representative for the system and the following orders of magnitude have been considered:

- Density of the polymer melt ~10^3 kg m^{-3}
- Diameter of the reactor ~ 10^{-1} m
- Viscosity of the melt ~ 10^2 Pa s

Supplementary Note 3 | Scope of the CFD model.

The CFD model included a close replication of the vessel and stirrer geometries as it was developed in collaboration with the manufacturer. Rheological properties of the melt (**Fig.1 c**) were used in the calculations to accurately represent local viscosity variations and therefore obtain a realistic picture of the velocity field. The model also assumed the following simplifications, mainly to keep computation time under 24 h per case study, in view of the complexity of the system involving a catalytic reaction with multiphasic flow and a non-Newtonian fluid.

- The lack of an available kinetic description for polyolefin hydrogenolysis precluded from simulating the evolution of product distribution over time. Hence, the model accurately represents fluid dynamics as long as the average viscosity of the melt remains close to that of the molten plastic. The monitoring of the torque revealed relatively small torque variations of 20-30% over the course of a typical experiment where the yield to C_{45+} products remained over 30%, suggesting that even minor fractions of long chain hydrocarbons can dictate the motion of the melt until the final stages of the reaction.
- In view of the constraint of the reaction to a region close to the melt described by the millimeter scale (**Supplementary Note 5**), the model did not include H_2 diffusion into the melt.
- Simulations were performed either considering H_2-melt or catalyst particles-H_2-melt phases as described in **Methods**.

Supplementary Note 4 | Estimation of penetration depth of polymers into large pores.

For the case of characteristic pore lengths in the order of micrometres, typically encountered in shaped catalysts,[3] a microscopic description of the capillary penetration of the molten polymer using the Washburn equation could be appropriate (Equation S6).[4]

$$\lambda = \sqrt{\frac{\gamma \cos(\phi) r t}{2\mu}} \quad \text{(S6)}$$

Modified versions of the Washburn equation are needed to describe the behavior of molten polymers at the nanoscale, which, nonetheless, converge to Equation S6 in the case of micrometer or larger scales.[5,6] According to it, the penetration length, λ of a liquid into a pore of radius r with a contact angle ϕ, and a surface tension γ has the functional dependency $\lambda \propto (t \cdot r \cdot \mu^{-1})^{0.5}$, where t represents time. Taking a surface tension[7] of 35 mJ m^{-2} and assuming a contact angle of 45° between the melt and catalyst particles,[5] the Washburn equation predicts a characteristic time of ca. 0.1 s for high M_w polyolefins to penetrate a pore with $r = 10^{-6}$ m even when considering viscosities under unstirred conditions (*i.e.*, zero shear rate, **Fig. 1**). This small characteristic time suggests that, even in the case of relatively large error in the estimation of the equation parameters, catalysts typically used in industry, comprising an active phase in the form of particles and a binder among them and showing intraparticle pore sizes commonly in the micrometre scale,[3] could be successfully used for processing of consumer grade polyolefins. However, this estimation seems at odds with recent visualization on the lack of accessibility of PP with high M_w into commercial fluid catalytic cracking catalyst particles,[8] hinting at further phenomena at play requiring further studies.

Supplementary Note 5 | Estimation of characteristic dimension of polymer molecules.

The maximum length of a representative chain of $HDPE_{200}$ is *ca.* 3 µm, if modelled as $C_{20000}H_{40000}$ ($M_w \sim 200$ kDa) and considering a C-C bond distance of $\delta = 0.154$ nm.[16] However, the numerous internal spatial degrees of freedom such as rotation around each C–C bond makes it is uncommon to observe it in a fully extended state. The typical dimension (Λ) for folded polymer chains in a uniform melt can be deduced from the Freely Jointed Chain model,[17] predicting $\Lambda = \Gamma^{0.5} \cdot \delta$, were Γ represents the number of C-C bonds in the backbone. This model provides a minimum boundary for Λ in our conditions, since polymer chains tend to unfold under shear stress.[18] This model thus predicts a very mild dependency of the typical chain size with M_w, resulting in $\Lambda \sim 22$ nm for $HDPE_{200}$.[19]

Supplementary Note 6 | Estimation of hydrogen diffusivities for melts with varying viscosity. The diffusivity of a species *i* in a liquid medium is usually inversely correlated to the viscosity of the medium, as predicted by the Stokes-Einstein equation. However, it was not possible to find correlations for molten polymers and H_2, so we used an experimental correlation of the type $D_i = K \exp(1/M_w)$, where K is a constant dependent on the diffusing species *i* and M_w the molecular weight of the melt.[20] Based on it, it was possible to estimate $D_{H2,\ HDPE20} = 3\ 10^{-8}$ m^2 s^{-1} and $D_{H2,HDPE2} = 5\ 10^{-8}$ m^2 s^{-1}, and thus simulate the H_2 profile for the case when the average chain length corresponds to 1/10 and 1/100 of the initial one provided in **Supplementary Fig. 7**. These larger diffusivities compared to $HDPE_{200}$ lead to a deeper penetration of H_2 but still far from equilibrium, even in the absence of reaction.

Supplementary Note 7 | Estimation of typical scale lengths of the reaction front.

The penetration and reaction of hydrogen into the melt upon contact with the catalyst particles was modeled assuming the diffusional transport of hydrogen into the melt and its homogeneous reaction in a film according to the thin film theory used to describe fluid-fluid reactions.[9] Pseudo first-order kinetics were assumed, where the volumetric reaction rate is given by $r = k_r c_{H2}$. This enabled the prediction of the evolution of c_{H2} with z, being z the coordinate penetrating into the melt with zero value at the H_2-melt interface. Under these conditions, the following mass balance holds under steady-state conditions (Equation S7):

$$D_{H2}\frac{d^2 c_{H2}}{dz^2} = k_r c_{H2} \tag{S7}$$

with boundary conditions:

$$c_{H2} = c_{H2,int} = \frac{p_{H2}}{H_{H2}} \qquad \text{at} \qquad z = 0 \text{ (interface)}$$

$$c_{H2} = 0 \qquad \textit{at} \qquad z = \infty \text{ (bulk)}$$

where equilibrium at the interface reflected by application of the Henry's law has been assumed. Solving this differential equation enables describing the decay of c_{H2} in the melt (Equation S8).

$$c_{H2} = c_{H2,\,int}\exp\left(-z\sqrt{\frac{k_r}{D_{H2}}}\right) \tag{S8}$$

The estimation of $c_{H2,int}$ and k_r contained in Equation S8 led different profiles depicted in **Fig. 2b**. The Henry's coefficient under operating conditions could be estimated[10] to be $H_{H2} \approx 0.4\cdot10^5$ mol_{H2} m^{-3} Pa^{-1}. From this and considering that $p_{H2} = 20\cdot10^5$ Pa under standard testing conditions, the concentration of H_2 at the interface is $c_{H2,int} \approx 50$ mol m^{-3}. The diffusivity of H_2 in molten polyolefins is not available in the open literature for temperatures above 500 K. We then used a slightly larger value of $D_{H2} = 1\cdot10^{-8}$ m^2 s^{-1} compared to measurements and simulations for another available small molecule like methane under similar conditions ($D_{CH4} = 4\cdot10^{-9}$-$9\cdot10^{-9}$ m^2 s^{-1}).[11,12]

For the calculation of the rate constant k_r, we estimated r based on the following assumptions:

- We modelled $HDPE_{200}$ conservatively as $C_{10000}H_{20000}$, which gives a molecular weight of 105 kDa, to account for variability in chain lengths present in $HDPE_{200}$ and assure a lower bound for r.
- Assumed its full conversion into $C_{50}H_{100}$ (included in the range of products denoted as residue in the manuscript), which also contributes to find a lower bound for the consumption of hydrogen, as all tests provided significant yields of C_1-C_{45} products.

- With this data we calculated the consumption of hydrogen per unit time, considering that the full transformation of 1 mol of $C_{10000}H_{15000}$ into $C_{50}H_{100}$ requires 200 moles of H_2. Considering the amount of plastic processed (0.5 g) and 4 h as reaction time, the hydrogen consumption resulted to be $6 \cdot 10^{-7}$ mol_{H2} s^{-1}. This value is in agreement with other reports.[13]
- Considering the volume of the melt ($4 \cdot 10^{-6}$ m^3), $r = 0.15$ mol_{H2} s^{-1} m^{-3} and therefore $k_r = 0.15/50 = 3 \cdot 10^{-3}$ s^{-1}. This value, according to Equation S8 and **Fig. 2b**, indicates that the concentration of hydrogen decays below 10% of the concentration at the interface within ca. 8 mm. If the target product is changed to $C_{20}H_{40}$ as more representative according to observed product distributions (**Supplementary Tables 5,6**), then $k_r \approx 10^{-2}$ s^{-1}, whereas the extreme case of full conversion into methane would have rendered $k_r \approx 0.15$ s^{-1}. Following this approach, the gray region in **Fig. 2b** indicating the regions for poorly and highly active catalysts could be found, evidencing that the reaction is mostly confined to the first millimeters of the melt. Using these parameters and a estimation for the mass transfer coefficient of hydrogen[10] of $k_{H2} = 10^{-6}$ m s^{-1}, a value of 5 was calculated for the Hatta number (ratio between the observed reaction rate and diffusion rate for hydrogen, Equation S9), reinforcing the assumption of the reaction be mostly confined to the vicinity of the H_2-melt interface.

$$\mathrm{Ha} = \frac{\sqrt{k_r D_{H_2}}}{k_{H_2}} \quad \text{(S9)}$$

Supplementary Note 8 | Influence of catalyst particle density in their motion.

A first factor to analyze the motion of the particles is the relative density between the melt (910 kg m^{-3} for PP_{340} and 1000 kg m^{-3} for $HDPE_{200}$)[14] and catalyst particles, since values below that of the molten polymer (*e.g.*, some zeolites) could help particles reach the top of the melt more easily. In most cases, like metal oxides or carbonaceous materials, catalyst particles show a larger density than the molten polymer ($\rho_p/\rho_m \sim 4$ in this study). However, the estimated values for the Archimedes number (Ar), giving the ratio between gravitational and viscous forces (Equation S10), varies from 10^{-8} (dense materials) to 10^{-7} (less dense materials) for typical sieve fractions in the order of 10^{-1} mm. This implies that the density of the catalyst is expected to play a negligible role in the motion of particles.

$$\mathrm{Ar} = \frac{\mathrm{g} d_\mathrm{p}^{\,3} \rho_\mathrm{m} (\rho_\mathrm{p} - \rho_\mathrm{m})}{\mu^2} \qquad \text{(S10)}$$

Supplementary Note 9 | Characterization of catalyst particle circulation in the vessel.

Increase particle circulation increases conversion as exposes particles more often to the reactive environment located at the vicinity of the H_2-melt interface (**Supplementary Note 3**). The z-Reynolds number of particles, defined using the velocity along the z-axis ($\mathrm{Re}_{\mathrm{p},z}$), is thus an appropriate non-dimensional descriptor for the vertical circulation of particles. Since particles predominantly follow the flow field of the polymer melt (**Supplementary Fig. 11**), characterized by vertical circulation patterns (**Fig. 3b** and **Supplementary Video 4**), high values of $\mathrm{Re}_{\mathrm{p},z}$ indicate frequent exposure of particles to the reaction front. This parameter is not observable as varies for each particle in space and time. The maximum value reached by $\mathrm{Re}_{\mathrm{p},z}$ ($\mathrm{Re}_{\mathrm{p},z,\mathrm{max}}$, Equation S11) is not observable either, but can be phenomenologically related to the tip velocity of the stirrer *via* simulations using the herein defined shape factor (K_s). This parameter thus describes the ability of different stirrer geometries to transfer the rotational kinetic energy of the stirrer into vertical kinetic energy of particles. **Extended Data Fig. 2** shows noticeable differences of up to two times among the three investigated stirrer geometries, with the impeller geometry showing the largest values for both polymers. In parallel, viscosity can be considered as constant in mechanical stirring at typical stirring rates and operation temperatures, as observed in **Fig. 1b**. $\mathrm{Re}_{\mathrm{p},z,\mathrm{max}}$ can thus be estimated based on observable variables (Equation S11), characterizing the rate of particle circulation, which is dictated by melt properties, stirring rate, and geometry of the stirrer and catalyst particles.

$$\mathrm{Re}_{\mathrm{p},z,\mathrm{max}} = \frac{\rho_\mathrm{m} v_{\mathrm{p},z,\mathrm{max}} d_\mathrm{p}}{\mu} \approx \frac{\rho_\mathrm{m} v_{\mathrm{p},z,\mathrm{max}} d_\mathrm{p}}{\bar{\mu}} = \frac{\rho_\mathrm{m} K_\mathrm{s} v_\mathrm{tip} d_\mathrm{p}}{\bar{\mu}} = \frac{\pi \rho_\mathrm{m} N K_\mathrm{s} D d_\mathrm{p}}{60\bar{\mu}} \qquad \text{(S11)}$$

Supplementary Note 10 | Relation between maximum vertical Re_p and performance.

Extended Data Fig. 3 shows the simulated distribution of particle vertical velocities for the three geometries (**Supplementary Table 7** and **Supplementary Fig. 13** for PP_{340}). The maximum vertical particle velocity in the reactor, $v_{p,z,max}$ achieved by propeller and impeller types was 50-60% larger than the turbine. The relation between particle circulation and performance could be quantitatively described after defining the maximum Reynolds number of catalyst particles along the *z*-axis ($Re_{p,z,max}$, Equation S11 and **Extended Data Table 2**) as a dimensionless descriptor for vertical catalyst particle circulation. This approach manages to retain the different features among stirrers observed in **Fig. 3**, where the propeller generally favours *xy*-velocities since it keeps particles separated in two distinct regions, whereas the impeller shows particle circulation in a narrow range of *z*-values.

$Re_{p,z,max}$ can thus be estimated from the melt properties, stirring rate, and particle and stirrer geometry after considering an average viscosity $\overline{\mu}$ (see **Fig. 1**) and through the herein defined shape factor, $K_s = v_{p,z,max}/v_{tip}$ (**Extended Data Table 2,** and **Supplementary Note 9),** which is dependent on the stirrer type and can be estimated from simulations and tabulated (**Extended Data Fig. 4** and **Supplementary Table 11**). This made possible to correlate performance parameters and $Re_{p,z,max}$ (**Extended Data Fig. 3**). The most noticeable result is the opposite trend of the selectivity to liquid products with increasing $Re_{p,z,max}$ for both polymers. In the case of $HDPE_{200}$, the use of turbines ($Re_{p,z,max} \sim 0.1\cdot10^{-4}$) is recommended, whereas for PP_{340}, impellers are more advantageous ($Re_{p,z,max} \sim 2.6\cdot10^{-4}$) to reach this fraction. This effect could be linked to the lower yields to C_1-C_{45} products obtained for the more chemically resistant PP_{340} (**Supplementary Table 6**). Stirrers promoting larger conversions by larger particle circulation (propeller, impeller) may facilitate progression of chain lengths towards the liquid range for this type of waste. A simple criterion through the estimation of $Re_{p,z,max}$ is thus available to predict conditions and stirrer geometries to tune selectivity for these two feedstocks.

Supplementary Note 11 | Characterization of the hydrogen-melt interface.

Since the reaction is predominantly constrained to the vicinity of the hydrogen-melt interface (**Supplementary Note 7**), effective catalyst testing requires increasing through stirring the magnitude of this interface (which equals the cross section of the vessel under no stirring). This parameter is not experimentally attainable. We estimated the relative efficiency of different stirring configurations via CFD simulations after defining the hydrogen fraction (χ_{H2}) in a volume close to the H_2-polymer melt as a proxy for the hydrogen-melt interface magnitude (see **Methods** for more details). The magnitude of this interface can be related to the degree of turbulence, and therefore average Reynolds number, which physically reflects on the hills and valleys formed under operation (**Fig. 4b,c**). However, the average Reynolds number close to the interface cannot be easily determined. Nevertheless, it can be inversely correlated to the power number (N_p, defined in Equation S12) regardless of the stirrer geometry for the case of Re < 10 (laminar flow),[1,15] a threshold that is two orders of magnitude larger than maximum values presented in **Fig. 4d**. N_p is thus a more convenient descriptor for the magnitude of the hydrogen-melt interface. Given that the density of a polymer melt is *ca.* four orders of magnitude larger than that of hydrogen, the simplification described in Equation S12 can be applied and N_p calculated from observable variables and χ_{H2}.

$$N_p = \frac{2\pi N\tau}{60\bar{\rho}(N/60)^3D^5} = \frac{7200\pi\tau}{(\chi_{H2}\rho_{H2} + (1-\chi_{H2})\rho_m)N^2D^5} \approx \frac{7200\pi\tau}{(1-\chi_{H2})\rho_m N^2D^5} \quad \text{(S12)}$$

However, accurate torque (τ) monitoring or control is not currently a widely available feature in test benches. We employed the stirrer model of concentric cylinders described in the Equations S1-S3 to relate the torque with the geometry of the stirrer and viscosity, arriving at Equation S13, where the assumption of a homogenous value of viscosity independent of stirring rate and temperature (**Fig. 1c**) under typical conditions was made.

$$N_p = \frac{\pi^2 LD^2\bar{\mu}}{(1-\chi_{H2})\rho_m N^2D^5}\cdot\frac{14400\pi ND^2D_r^2}{D^2(D_r^2-D^2)} = \frac{14400\pi^3\bar{\mu}D_r^2L}{(1-\chi_{H2})\rho_m ND^3(D_r^2-D^2)} \quad \text{(S13)}$$

All variables in Equation S13 are observable or part of the design process, except χ_{H2}. CFD simulations enabled to correlate χ_{H2} and N_p (**Extended Data Fig. 5**) and revealed its dependency with stirrer and plastic type, while displaying a general tendency to plateau in a wide range of stirring rates.

These results may allow the estimation of N_p and χ_{H2} based on an iterative approach. Nevertheless, χ_{H2} = 0.20-0.22 for a wide range of stirring rates, which allows the direct estimation of N_p with a sufficient accuracy and thus calculation of the effectiveness factor from **Fig. 4d** and **Fig. 5**. However, it is relevant to notice that simulations did not account for the evolution of product distribution toward lighter alkanes, making the overall viscosity decrease over time in real operation, and thus expectedly increasing the degree of turbulence for a

constant stirring rate as the reaction progresses. This reasoning may be behind the shift in optimal stirring rates observed between experimental results (**Fig. 4a**) and simulations (**Extended Data Figure 5**). With this in mind, we calculated N_p values in **Fig. 4d** using *N* values extracted from **Fig. 4a** and corresponding χ_{H2} from nominal stirring rates in **Extended Data Fig. 5** to offer a valuable criterion to catalysis practitioners.

The criterion presents a range of N_p for which the effectiveness factor is maximized for both plastics. According to Equation S13, it is then possible to select different combinations of stirring rates, stirrer, and vessel geometry to achieve optimal N_p ranges available in **Fig. 4d** and **Fig. 5**.

Supplementary Note 12 | Optimal stirring rates in experiments and simulations.

It is relevant to note that CFD simulations used herein consider the properties of the polymer melts and therefore do not consider the appearance of products with different physical properties as the reaction progresses (**Supplementary Note 3**). Since viscosity decreases with the chain length, it is expected that simulated optimal power numbers (**Extended Data Fig. 5**) to maximize χ_{H2} be larger than optimal experimental ones (**Fig. 4a**). This was supported by CFD simulations (**Supplementary Fig. 16**) considering eicosane (one of the lightest liquid fraction possible), which yielded a deeper penetration of H_2 in the melt (and therefore larger χ_{H2}) under similar stirring rates due to the formation of bubbles. This technicality is, nevertheless, of secondary relevance, as it only influences the determination of χ_{H2}, which displays values around 0.2 for a broad range of stirring rates (**Extended Data Fig. 5**).

Supplementary Figures

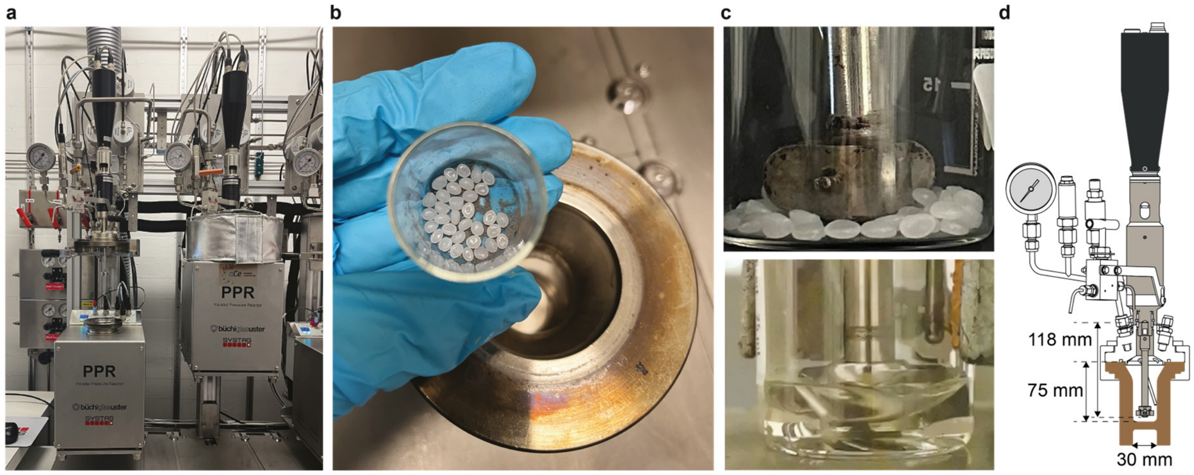

Supplementary Fig. 1 | Experimental setup and reactor configuration. **a** View of two of the parallel pressurized reactors. **b** Glass inset containing plastic beads to be placed inside the stainless-steel reactor. **c** Placement and operation of the stirrer inside the reactor inset (**Supplementary Video 3**). **d** Representative sketch of a single reactor showing key dimensions.

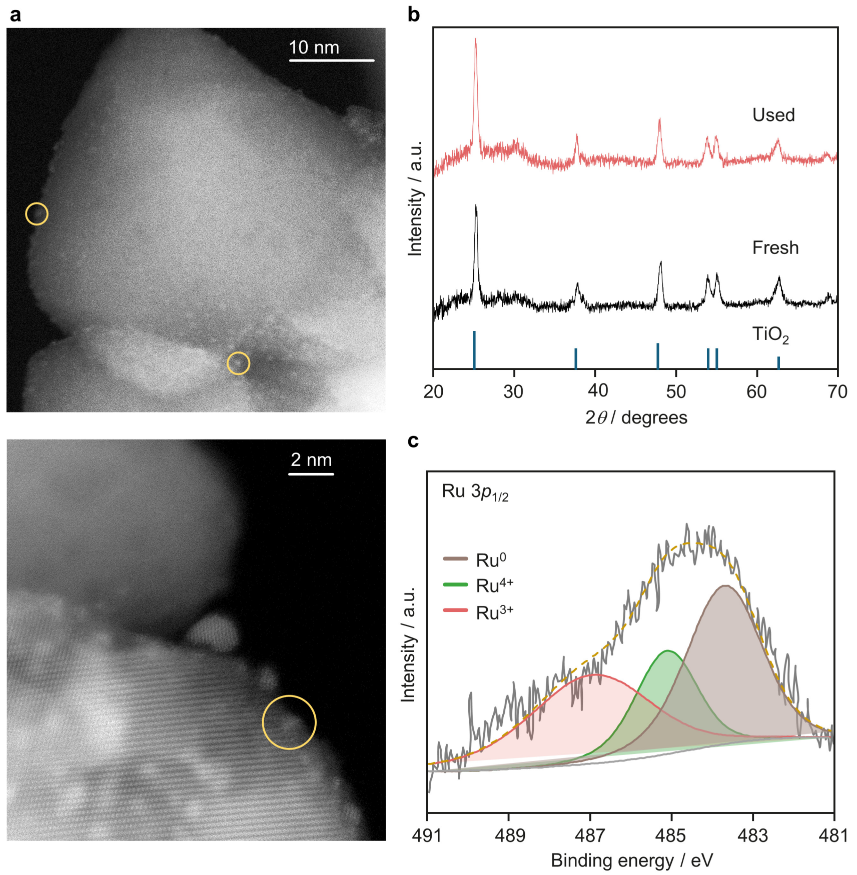

Supplementary Fig. 2 | Physicochemical characterization of the reference Ru/TiO_2 catalyst.[21] **a** Representative HAADF-TEM micrographs from used samples showing nanometric-sized Ru particles (exemplified in yellow circles) on titania (anatase). **b** X-ray diffractograms from representative fresh and used samples. **c** X-ray photoelectron spectrum of a representative used sample with signals used for deconvolution indicated.

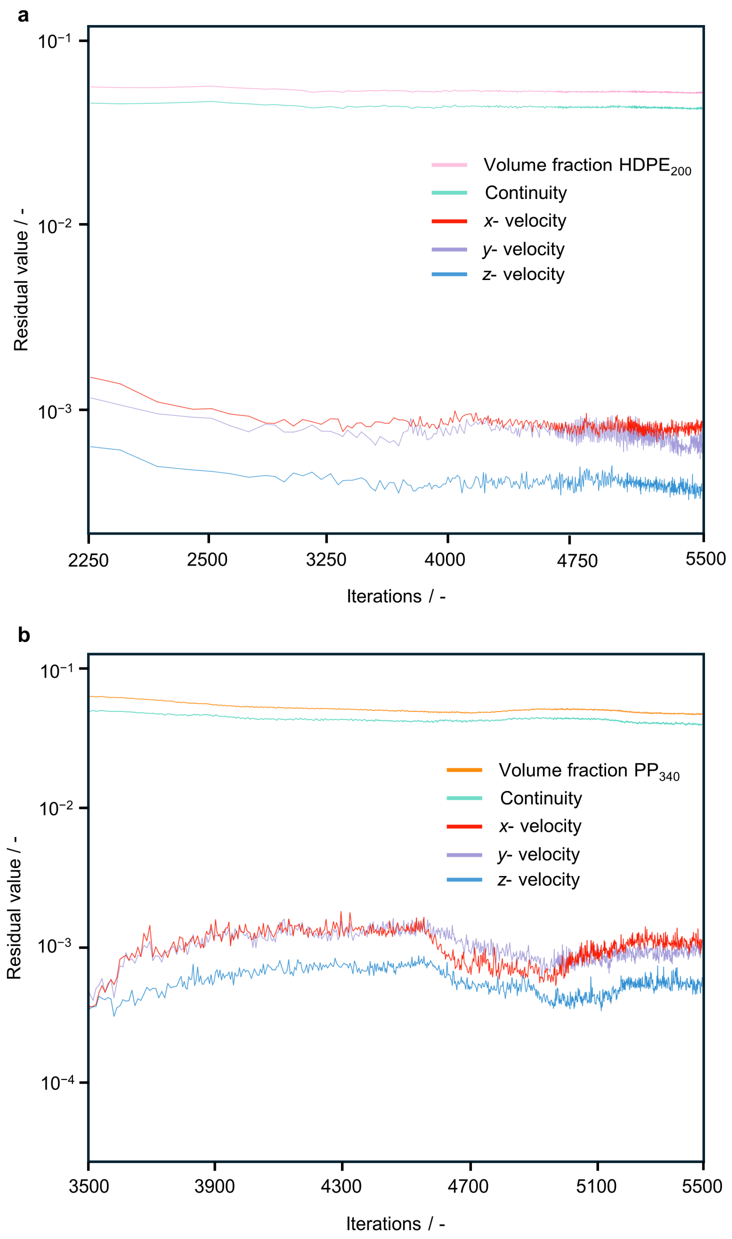

Supplementary Fig. 3 | Representative convergence plots for Volume-of-Fluid simulations. Residual values at the latest stages of computation for **a** $HDPE_{200}$ **b** PP_{340}. Cases shown: two-phase simulation with stirring rate = 750 rpm.

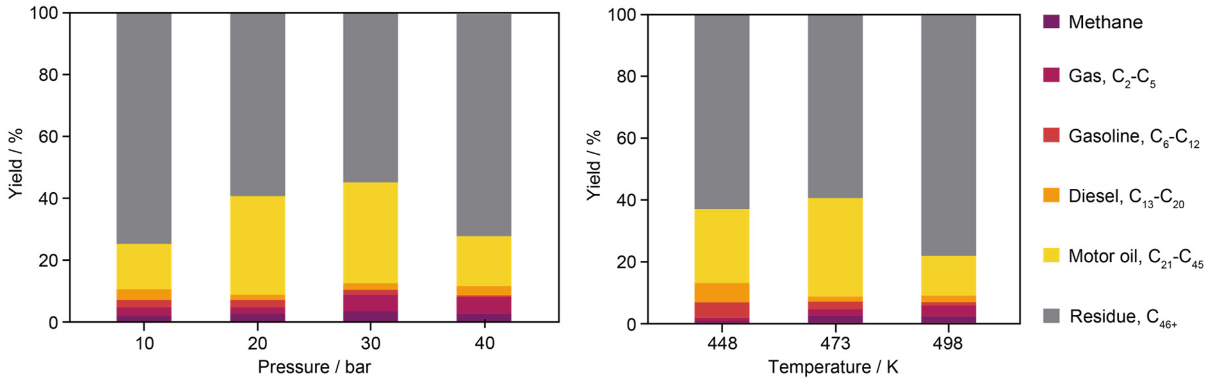

Supplementary Fig. 4 | Catalytic performance for hydrogenolysis of PP_{340} at different pressures and temperatures. Product distribution under different hydrogen pressure (left) and temperature (right). Reaction conditions: catalyst/plastic = 0.05, d_p = 0.0-0.2 mm, stirrer = propeller, stirring rate = 750 rpm, t = 4 h.

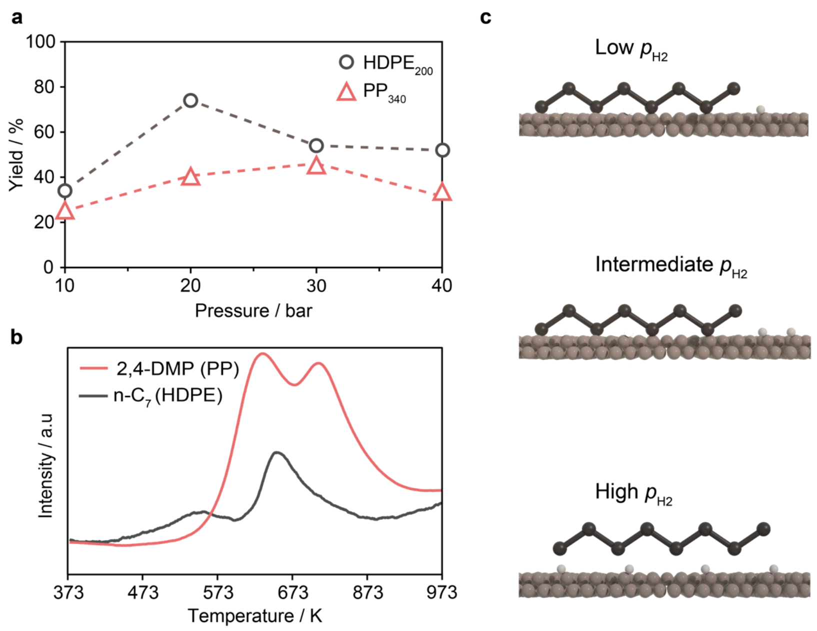

Supplementary Fig. 5 | Competitive adsorption of hydrogen and polyolefins. a Yield to C_1-C_{45} products under different pressures obtained from **Extended Data Fig. 2** and **Suplemementary Fig. 4** for $HDPE_{200}$ and PP_{340}, respectively. Reaction conditions: T = 498 K, catalyst/plastic = 0.05, d_p = 0.0-0.2 mm, stirrer = propeller, stirring rate = 750 rpm. **b** Temperature-programmed desorption profiles of *n*-heptane and 2,4-dimethylpentane over the Ru/TiO_2 catalyst as surrogate molecules for $HDPE_{200}$ and PP_{340}, respectively, showing stronger adsorption energies for the PP_{340} surrogate. This supports a competitive adsorption between the polyolefin and H_2 giving rise to shifted optimal H_2 pressures in **a**. **c** Schematic visualization of the inhibition of polyolefin adsorption under increasing hydrogen pressure.

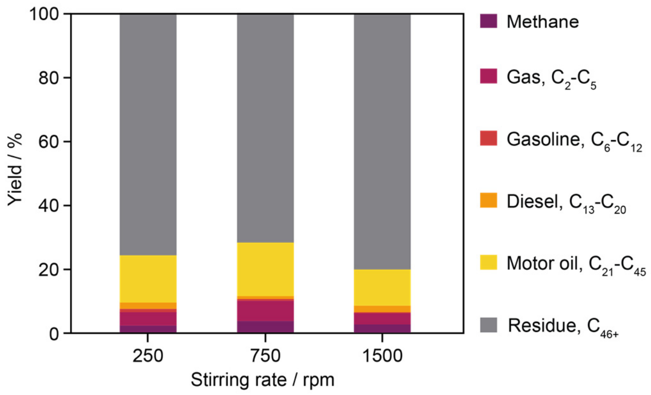

Supplementary Fig. 6| Catalytic performance for hydrogenolysis of $HDPE_{200}$ using d_p = 0.4-0.6 mm at different stirring rates. Reaction conditions: T = 498 K, p_{H_2} = 20 bar, catalyst/plastic = 0.05, stirrer = impeller, t = 4 h.

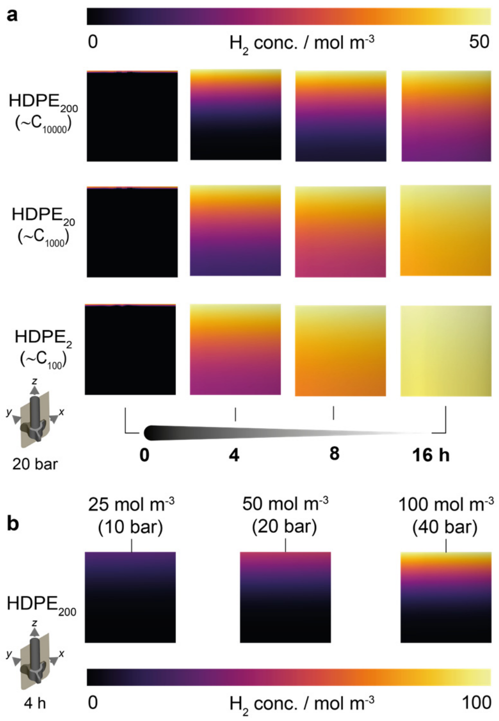

Supplementary Fig. 7 | Simulation of H_2 diffusion in a plastic melt. Simulated concentration profile of hydrogen in the *z*-*x* plane owing to its diffusion into stagnant molten $HDPE_{200}$ with varying **a** chain length and time and **b** in contact with different hydrogen pressures after 4 h. Simulation parameters for **a** provided in **Supplementary Note 6** and the provided concentration at the interface in **b** calculated from Henry's law (**Supplementary Note 7**). T = 498 K.

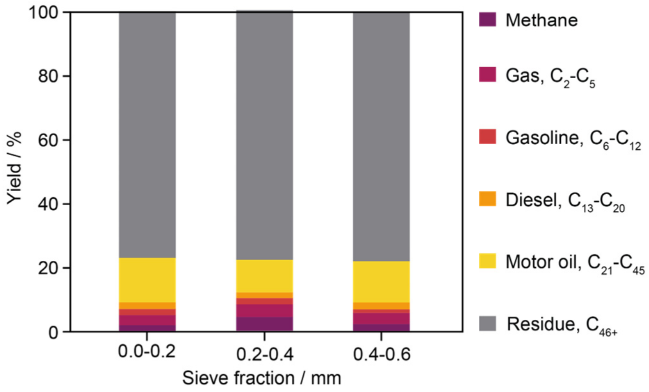

Supplementary Fig. 8 | Catalytic performance for hydrogenolysis of PP_{340} using different catalyst sieve fractions. Reaction conditions: T = 498 K, p_{H_2} = 20 bar, catalyst/plastic = 0.05, stirrer = impeller, stirring rate = 750 rpm, t = 4 h.

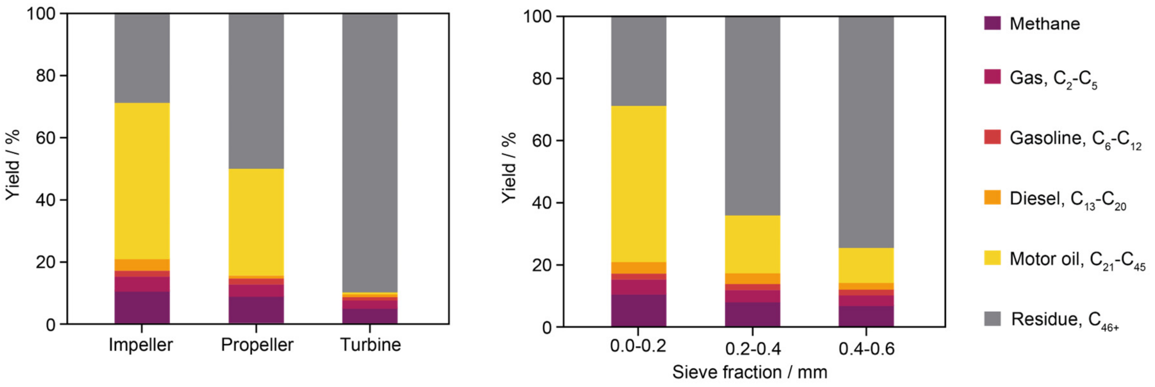

Supplementary Fig. 9 | Catalytic performance for hydrogenolysis of PP_{12} using different stirrers and catalyst sieve fractions. Reaction conditions: T = 498 K, p_{H_2} = 20 bar, catalyst/plastic = 0.05, stirrer = impeller (right), stirring rate = 750 rpm, t = 4 h.

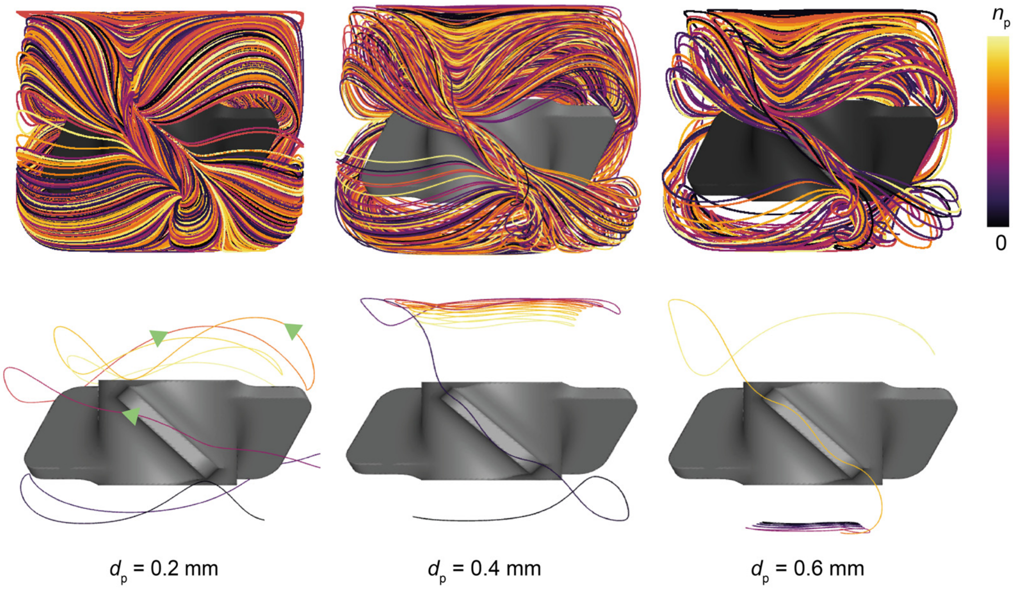

Supplementary Fig. 10 | Particle trajectories for different catalyst particle sizes. Simulated particles trajectories once steady state is reached for n_p =100-200 particles (top) and initial trajectories for a representative single particle (bottom) within a single-phase melt of $HDPE_{200}$ across different catalyst particle sizes. Simulated conditions: T = 498 K, p_{H_2} = 20 bar, catalyst/plastic = 0.05, stirrer = propeller, stirring rate = 750 rpm.

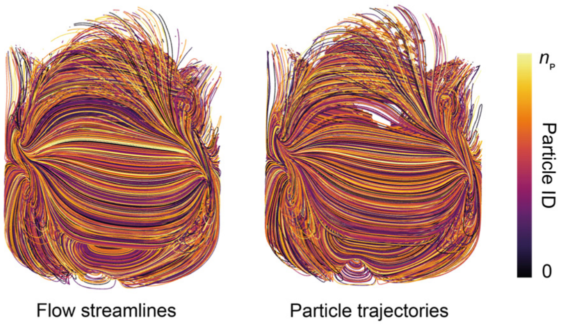

Supplementary Fig. 11 | Comparison between flow streamlines and particle trajectories. 3-phase CFD simulation comparing flow streamlines and particle trajectories (d_p = 0.2 mm) for the case of $HDPE_{200}$. n_p = 200 is the total number of particles included in the simulation. Simulated conditions: T = 498 K, p_{H_2} = 20 bar, catalyst/plastic = 0.05, stirrer = impeller, stirring rate = 750 rpm.

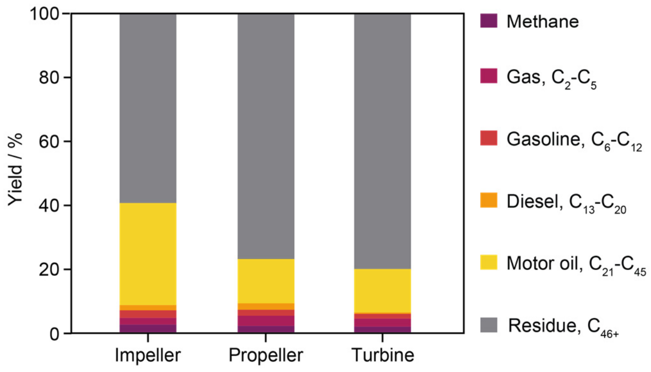

Supplementary Fig. 12 | Catalytic performance for hydrogenolysis of PP_{340} with different stirrers. Product distribution obtained using impeller, propeller, and turbine types. T = 498 K, p_{H_2} = 20 bar, catalyst/plastic = 0.05, d_p = 0.0-0.2 mm, stirring rate = 750 rpm, t = 4 h.

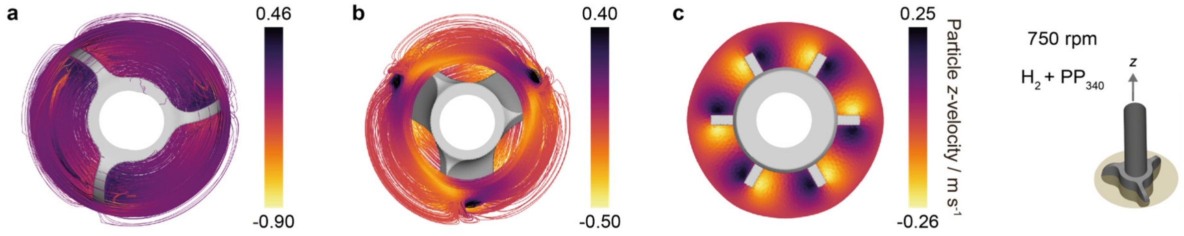

Supplementary Fig. 13 | Distribution of *z*-velocities of catalyst particles in molten PP_{340}. Top view of simulated distribution of particle velocities along the *z* axis once steady state is reached for **a** impeller **b** propeller, and **c** turbine stirrers. Simulated conditions: T = 498 K, p_{H2} = 20 bar, catalyst/plastic = 0.05, d_p = 0.2 mm, stirring rate = 750 rpm.

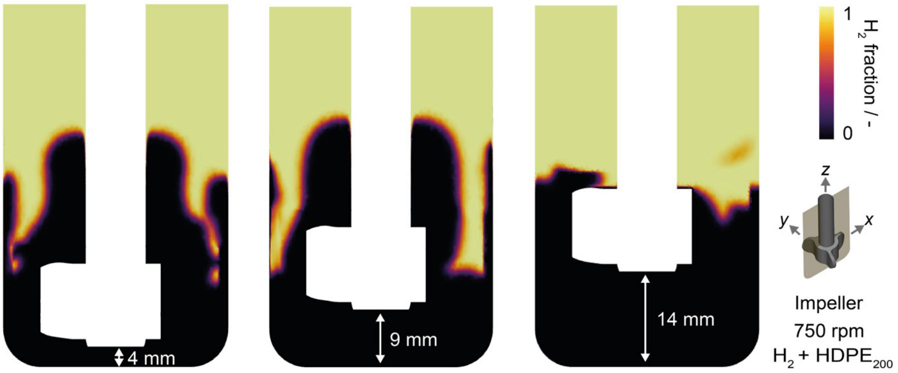

Supplementary Fig. 14 | Influence of stirrer vertical position on the hydrogen fraction. Evolution of the hydrogen fraction with an increasingly higher location of the stirrer for $HDPE_{200}$. Simulated conditions: T = 498 K, p_{H_2} = 20 bar, stirrer = impeller, stirring rate = 750 rpm.

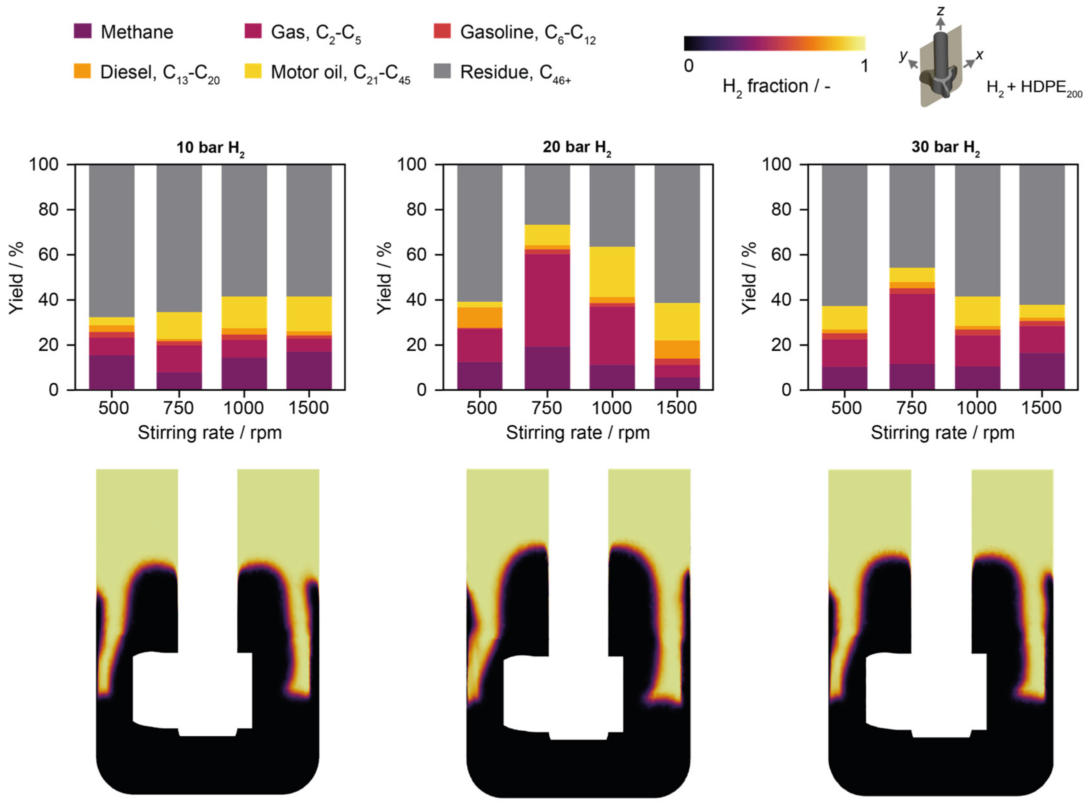

Supplementary Fig. 15 | Catalytic performance for hydrogenolysis of $HDPE_{200}$ at different pressures. Product distribution under different hydrogen pressure and corresponding 2-phase CFD simulations of the hydrogen fraction underneath. Reaction and simulated conditions: catalyst/plastic = 0.05, d_p = 0.0-0.2 mm, stirrer = impeller, t = 4 h.

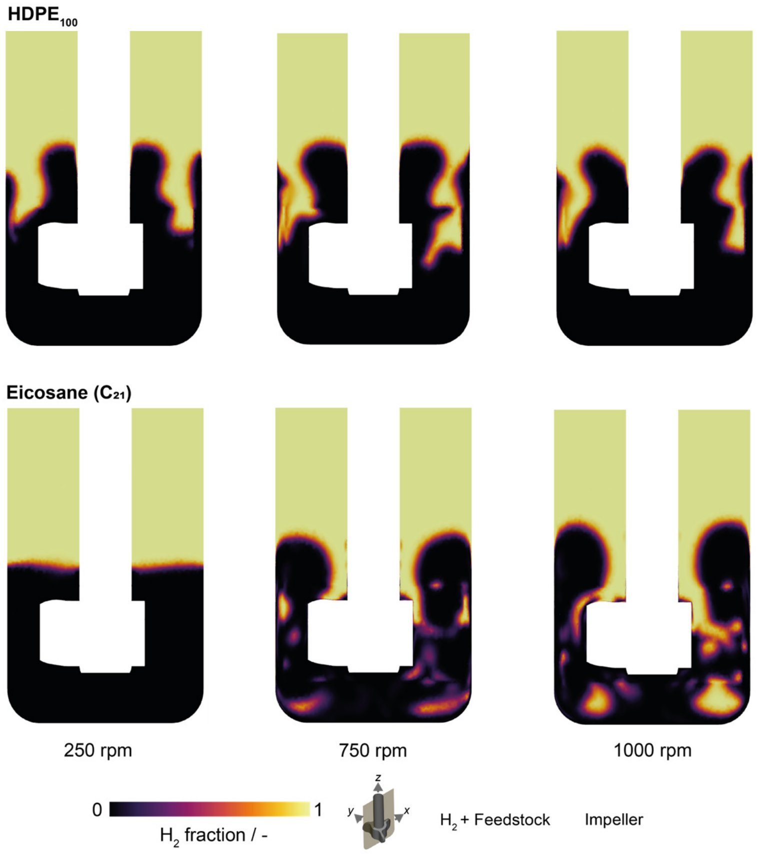

Supplementary Fig. 16 | Influence of stirring rate on hydrogen fraction for HDPE$_{100}$ and eicosane. 2-phase CFD simulations of the evolution of the hydrogen fraction with stirring rate for HDPE$_{100}$ and eicosane. T = 498 K, p_{H_2} = 20 bar, stirrer = impeller, stirring rate = 750 rpm.

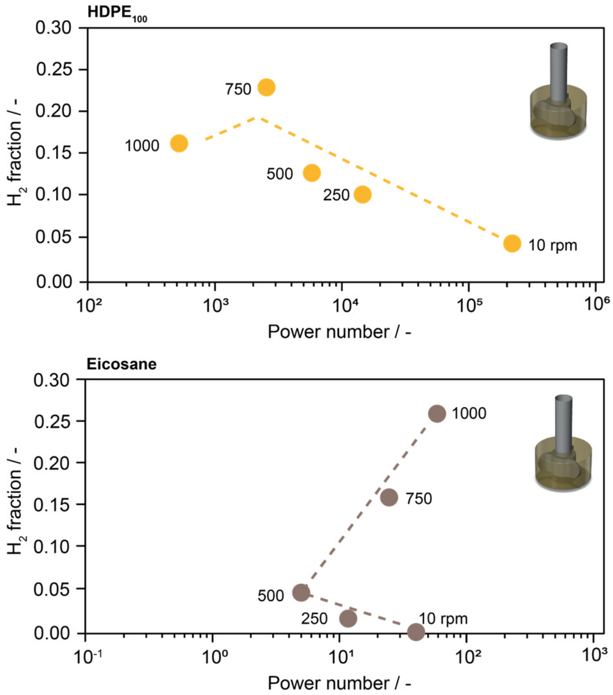

Supplementary Fig. 17 | Simulated hydrogen fractions for $HDPE_{100}$ and eicosane. Variation of the hydrogen fraction in the melt volume with power number at different stirring rates calculated from CFD simulations corresponding to those shown in **Fig. 6** for the case of impeller for $HDPE_{100}$ and eicosane**.** Values can be found in **Supplementary Table 13**.

Supplementary Tables

Supplementary Table 1 | Polyolefins used in this study with commercially reported physicochemical properties.

**Characteristic**	**$HDPE_{200}$**	**$HDPE_{100}$**	**PP_{340}**	**PP_{12}**
Supplier	Sigma Aldrich	Sigma Aldrich	Sigma Aldrich	Sigma Aldrich
Product ID	547999-1KG	427985-1KG	427861-1KG	428116-1KG
Weight average molecular weight / kDa	~200	~100	~340	~12
Melt flow index / kg min^{-1}	2.2	12	4	N.A
Melting point / K	413	413	433	433
Main applications	Bottle caps	Injection molding	Automotive, joints	PP films

Supplementary Table 2 | Accumulated number of articles in the field of plastic hydrogenolysis or hydrocracking, counted with respect to the type of stirring and type of feedstock used (M_w > 100 kDa is labelled as commercial grade).

Year	Mechanical stirring	Magnetic stirring	No/unspecified mixing	Commercial grade	Model/lab grade
2019	3	1	6	1	4
2020	6	3	10	5	9
2021	11	15	14	14	21
2022	17	26	19	19	38
2023	30	42	35	35	74

Supplementary Table 3 | Viscosity measured at different temperatures from rheological analysis of $HDPE_{200}$.

μ / s^{-1}	μ / Pa s (473 K)	μ / Pa s (498 K)	μ / Pa s (523 K)
0.001	5070	2835	1481
0.002	5212	2899	1831
0.005	5267	2956	1941
0.010	5291	2985	1989
0.021	5273	2994	1992
0.046	5232	2973	1954
0.100	5094	2859	1839
0.215	4759	2610	1579
0.464	4291	2331	1189
1.000	3724	1967	699
2.154	3125	1581	774
4.640	2490	1236	593
10.009	1354	923	264
21.548	662	519	166
49.964	143	163	163

Supplementary Table 4 | Viscosity obtained at different temperatures from rheological analysis of PP_{340}.

**μ / s^{-1}**	**μ / Pa s (473 K)**	**μ / Pa s (498 K)**	**μ / Pa s (523 K)**
0.001	6827	3762	2317
0.002	6926	4173	2366
0.005	6970	4367	2381
0.010	6952	4429	2396
0.021	6929	4453	2393
0.046	6853	4431	2380
0.100	6614	4314	2337
0.215	6102	4025	2233
0.464	5433	3629	2065
1.000	4611	3143	1849
2.154	3656	2665	1598
4.640	2677	2009	1310
10.009	1246	687	281
21.548	382	310	83
49.964	139	84	104

Supplementary Table 5 | Catalytic performance for hydrogenolysis of $HDPE_{200}$ under all tested configurations.

T / K	p_{H_2} / bar	*N* / rpm	Sieve fraction / mm	Stirrer type	Methane / %	Gas C_2-C_5 / %	Gasoline C_6-C_{12} / %	Diesel C_{13}-C_{20} / %	Motor oil C_{21}-C_{45} / %	Residue C_{46+} / %
498	20	750	0.0-0.2	Impeller	21.4	39.0	2.9	2.1	8.9	25.7
498	20	750	0.0-0.2	Propeller	15.2	29.1	2.1	6.7	15.2	32.0
498	20	750	0.0-0.2	Turbine	11.5	20.8	1.0	2.7	28.8	35.3
498	20	750	0.2-0.4	Propeller	7.1	13.7	0.9	0.5	17.1	60.4
498	20	750	0.4-0.6	Propeller	3.6	6.3	0.6	0.9	16.7	71.8
498	20	250	0.4-0.6	Impeller	2.76	3.2	1.5	2.5	15.3	74.4
498	20	1500	0.4-0.6	Impeller	2.94	3.1	1.4	2.1	10.9	79.3
473	20	750	0.0-0.2	Impeller	3.3	8.1	3.9	2.6	21.2	60.9
498	10	250	0.0-0.2	Impeller	15.6	8.0	2.6	3.1	3.7	69.4
498	10	750	0.0-0.2	Impeller	7.9	11.8	1.8	0.9	11.9	65.7
498	10	1000	0.0-0.2	Impeller	14.3	7.7	2.4	2.8	15.2	57.3
498	10	1500	0.0-0.2	Impeller	16.7	5.7	1.5	1.8	15.5	58.6
498	30	250	0.0-0.2	Impeller	10.5	12.2	2.7	1.6	10.4	62.6
498	30	750	0.0-0.2	Impeller	21.4	39.0	2.5	2.7	6.4	45.7
498	30	1000	0.0-0.2	Impeller	10.4	13.7	2.6	1.6	10.3	61.2
498	30	1500	0.0-0.2	Impeller	16.4	11.8	2.3	1.4	11.5	56.4
448	20	750	0.0-0.2	Impeller	11.6	31.1	0.7	1.3	18.5	69.7
498	20	0	0.0-0.2	Impeller	2.8	4.2	n.d.*	n.d.	n.d.	93.0
498	20	250	0.0-0.2	Impeller	12.4	14.6	0.5	9.1	2.4	60.9

(Continues in the next page)

(Continues from previous page)

498	20	500	0.0-0.2	Impeller	7.0	17.5	0.9	2.8	21.8	49.9
498	20	1000	0.0-0.2	Impeller	11.2	25.8	1.6	2.7	22.2	36.1
498	20	1500	0.0-0.2	Impeller	5.5	5.5	3.8	9.2	14.9	60.9

*n.d: not detected

Supplementary Table 6 | Catalytic performance for hydrogenolysis of PP_{340} under all tested configurations.

T / K	p_{H_2} / bar	N / rpm	Sieve fraction / mm	Stirrer type	Methane / %	Gas C_2-C_5 / %	Gasoline C_6-C_{12} / %	Diesel C_{13}-C_{20} / %	Motor oil C_{21}-C_{45} / %	Residue C_{46+} / %
498	20	750	0.0-0.2	Impeller	4.9	2.8	2.4	1.6	31.8	59.2
498	20	750	0.0-0.2	Propeller	5.2	2.1	1.8	2.0	13.9	76.8
498	20	750	0.0-0.2	Turbine	4.4	1.8	1.5	0.5	13.6	80.1
498	20	750	0.2-0.4	Propeller	8.3	4.3	1.9	1.8	10.1	77.8
498	20	750	0.4-0.6	Propeller	6.0	2.5	1.1	2.1	12.8	77.8
473	20	750	0.0-0.2	Impeller	1.6	5.1	3.6	1.5	13.5	71.6
498	10	750	0.0-0.2	Impeller	2.2	2.7	2.3	3.4	14.6	74.7
498	30	750	0.0-0.2	Impeller	2.8	6.2	1.6	2.1	32.6	54.8
498	40	750	0.0-0.2	Impeller	2.9	5.4	0.5	3.0	16.1	72.0
448	20	750	0.0-0.2	Impeller	0.5	1.6	0.9	1.6	14.0	80.9
498	20	0	0.0-0.2	Impeller	n.d.*	n.d.	n.d.	n.d.	n.d.	100.0
498	20	250	0.0-0.2	Impeller	2.3	2.7	0.7	0.8	16.4	77.1
498	20	500	0.0-0.2	Impeller	0.6	1.7	1.3	1.3	15.9	79.2
498	20	1000	0.0-0.2	Impeller	1.5	2.5	1.8	2.4	15.6	76.3
498	20	1500	0.0-0.2	Impeller	1.9	2.0	0.5	0.5	6.9	88.1

*n.d: not detected

Supplementary Table 7 | Single-phase simulation results for $HDPE_{200}$ and PP_{340} at different temperatures across the *x-y* plane passing through the equator of the stirrer blade. Simulated conditions: stirrer = propeller, stirring rate = 750 rpm.

**Polyolefin**	***T* / K**	**Avg. μ / Pa s**	**Avg. $Re \cdot 10^{-5}$ / -**
$HDPE_{200}$	498	549	49
$HDPE_{200}$	473	728	30
PP_{340}	498	448	38

Supplementary Table 8 | Catalytic performance for hydrogenolysis of $HDPE_{100}$, Eicosane, and PP_{12} under various tested configurations.

Reaction conditions: p_{H2} = 20 bar, T = 498 K, catalyst/plastic = 0.05.

**Feedstock**	***t* / *h***	***N* / rpm**	**Sieve fraction / mm**	**Stirrer type**	**Methane / %**	**Gas C_2-C_5 / %**	**Gasoline C_6-C_{12} / %**	**Diesel C_{13}-C_{20} / %**	**Motor oil C_{21}-C_{45} / %**	**Residue C_{46+} / %**
$HDPE_{100}$	2	0	0.0-0.2	Impeller	6.4	10.1	4.6	6.3	10.8	61.8
$HDPE_{100}$	2	500	0.0-0.2	Impeller	27.0	10.8	3.4	4.5	16.4	38.0
$HDPE_{100}$	2	750	0.0-0.2	Impeller	26.4	14.9	4.6	1.9	23.4	28.8
$HDPE_{100}$	2	1000	0.0-0.2	Impeller	25.7	13.0	6.1	4.1	14.1	37.0
Eicosane	2	0	0.0-0.2	Impeller	12.0	19.0	1.5	3.2	13.6	50.6
Eicosane	2	500	0.0-0.2	Impeller	54.1	35.5	10.1	0.0	n.d.*	0.4
Eicosane	2	750	0.0-0.2	Impeller	70.0	29.8	2.4	0.0	n.d.	n.d.
Eicosane	2	1000	0.0-0.2	Impeller	56.6	26.9	14.7	2.5	n.d.	n.d.
PP_{12}	4	750	0.0-0.2	Impeller	11.2	5.3	2.0	2.8	49.3	29.4
PP_{12}	4	750	0.0-0.2	Propeller	10.9	4.3	1.7	1.2	32.7	49.2
PP_{12}	4	750	0.0-0.2	Turbine	4.9	3.6	1.7	1.2	0.9	87.7
PP_{12}	4	750	0.2-0.4	Impeller	9.7	3.6	2.4	3.9	18.5	61.9
PP_{12}	4	750	0.4-0.6	Impeller	6.5	2.8	2.7	3.4	11.0	73.6

*n.d: not detected

Supplementary Table 9 | Cumulative scission events for hydrogenolysis of $HDPE_{200}$ for different catalyst sieve fractions and stirrer geometries. Reaction conditions: T = 498 K, p_{H2} = 20 bar, stirring rate = 750 rpm, catalyst/plastic = 0.05.

Sieve fraction / mm	Stirrer type	Scission events* / mmol
0.0-0.2	Impeller	11.7
0.0-0.2	Propeller	8.5
0.0-0.2	Turbine	7.8
0.2-0.4	Propeller	4.8
0.4-0.6	Propeller	1.5

*mmol of -CH_2- units cleaved

Supplementary Table 10 | Simulated maximum *z*-velocity of catalyst particles for hydrogenolysis of $HDPE_{2100}$ and PP_{340} for different stirrer types. Simulated conditions: d_p = 0.2 mm, stirring rate = 750 rpm.

**Polyolefin**	**Stirrer type**	**Max $v_{p,z}$ / m s^{-1}**
$HDPE_{200}$	Impeller	0.45
$HDPE_{200}$	Propeller	0.35
$HDPE_{200}$	Turbine	0.25
PP_{340}	Impeller	0.47
PP_{340}	Propeller	0.40
PP_{340}	Turbine	0.26

Supplementary Table 11 | Shape factors for the three stirrer types. Simulated conditions: catalyst/plastic = 0.05, d_p = 0.2 mm, stirring rate = 750 rpm, v_{tip} = 2.6 m s^{-1}.

**Polyolefin**	**Stirrer type**	**K_s / -**
$HDPE_{200}$	Impeller	0.17
$HDPE_{200}$	Propeller	0.13
$HDPE_{200}$	Turbine	0.09
PP_{340}	Impeller	0.17
PP_{340}	Propeller	0.15
PP_{340}	Turbine	0.09

Supplementary Table 12 | Simulated average power number and H_2 fraction for hydrogenolysis of $HDPE_{200}$ and PP_{340} for different stirrer types and stirring rates. Simulated conditions: T = 498 K, catalyst/plastic = 0.05, d_p = 0.2 mm.

**Polyolefin**	**Stirrer type**	***N* / rpm**	**$N_p \cdot 10^5$ / -**	**χ_{H2} / -**
$HDPE_{200}$	Impeller	10	24.96	0.06
$HDPE_{200}$	Impeller	250	1.89	0.22
$HDPE_{200}$	Impeller	500	0.53	0.22
$HDPE_{200}$	Impeller	750	0.26	0.23
$HDPE_{200}$	Impeller	1000	0.16	0.22
$HDPE_{200}$	Impeller	1500	0.08	0.22
$HDPE_{200}$	Impeller	1750	0.08	0.27
$HDPE_{200}$	Impeller	2500	0.02	0.15
$HDPE_{200}$	Propeller	10	18.39	0.04
$HDPE_{200}$	Propeller	250	0.29	0.14
$HDPE_{200}$	Propeller	750	0.11	0.22
$HDPE_{200}$	Propeller	1750	0.02	0.15
$HDPE_{200}$	Turbine	10	20.00	0.00
$HDPE_{200}$	Turbine	250	0.01	0.00
$HDPE_{200}$	Turbine	1250	0.00	0.00
$HDPE_{200}$	Turbine	1750	0.00	0.00
PP_{340}	Impeller	10	26.27	0.06
PP_{340}	Impeller	250	2.10	0.23
PP_{340}	Impeller	500	0.84	0.23
PP_{340}	Impeller	750	0.33	0.22
PP_{340}	Impeller	1000	0.16	0.21
PP_{340}	Impeller	1500	0.19	0.22
PP_{340}	Impeller	1750	0.07	0.20
PP_{340}	Propeller	10	17.08	0.04
PP_{340}	Propeller	250	0.50	0.20
PP_{340}	Propeller	750	0.11	0.21
PP_{340}	Propeller	1750	0.02	0.09
PP_{340}	Turbine	10	20.02	0.00
PP_{340}	Turbine	250	0.06	0.00
PP_{340}	Turbine	1750	0.00	0.00

Supplementary Table 13 | Simulated average power number and H_2 fraction for hydrogenolysis of $HDPE_{100}$ and eicosane. Simulated conditions: T = 498 K, catalyst/plastic = 0.05, d_p = 0.2 mm, stirrer = impeller.

Polyolefin	*N* / rpm	$N_p \cdot 10^5$ / -	χ_{H2} / -
$HDPE_{100}$	10	2.62	0.06
$HDPE_{100}$	250	0.15	0.1
$HDPE_{100}$	500	0.06	0.12
$HDPE_{100}$	750	0.03	0.22
$HDPE_{100}$	1000	0.006	0.15
Eicosane	10	0.0005	0.22
Eicosane	250	0.0001	0.27
Eicosane	500	0.00005	0.15
Eicosane	750	0.0002	0.04
Eicosane	1000	0.0006	0.14

Supplementary References

1. Hemrajani, R. R. & Tatterson, G. B. Mechanically stirred vessels. in *Handbook of Industrial Mixing* 345–390 (John Wiley & Sons, Inc., 2003).
2. Sinnott, R. & Towler, G. Equipment selection, specification and design. in *Chemical Engineering Series* (eds. Sinnott, R. & Towler, G.) 525–644 (Butterworth-Heinemann, 2020).
3. Mitchell, S., Michels, N.-L. & Pérez-Ramírez, J. From powder to technical body: the undervalued science of catalyst scale up. *Chem. Soc. Rev.* **42**, 6094–6112 (2013).
4. Washburn, E. W. Note on the dynamics of capillary flow. *Phys. Rev.* **17**, 273–283 (1921).
5. Yao, Y., Butt, H.-J., Floudas, G., Zhou, J. & Doi, M. Theory on capillary filling of polymer melts in nanopores. *Macromol. Rapid Commun.* **39**, 1800087 (2018).
6. Dimitrov, D. I., Milchev, A. & Binder, K. Capillary rise in nanopores: Molecular dynamics evidence for the Lucas-Washburn equation. *Phys. Rev. Lett.* **99**, 1–4 (2007).
7. Sauer, B. B. & Dee, G. T. Surface tensions of molten polymers as a function of molecular weight and temperature. *MRS Online Proc. Libr.* **248**, 441–446 (1991).
8. Rejman, S. *et al.* Transport limitations in polyolefin cracking at the single catalyst particle level. *Chem. Sci.* **14**, 10068–10080 (2023).
9. Levenspiel, O. Fluid-particle reaction kinetics. in *Chemical Reaction Engineering* 566–606 (John Wiley and Sons, New York, 1999).
10. Ge, J. & Peters, B. Mass transfer in catalytic depolymerization: External effectiveness factors and serendipitous processivity in stagnant and stirred melts. *Chem. Eng. J.* **466**, 143251 (2023).
11. Lundberg, J. L. Diffusivities and solubilities of methane in linear polyethylene melts. *J. Polym. Sci. Part A Gen. Pap.* **2**, 3925–3931 (1964).
12. Dutta, R. C. & Bhatia, S. K. Transport diffusion of light gases in polyethylene using atomistic simulations. *Langmuir* **33**, 936–946 (2017).
13. Tennakoon, A. *et al.* Catalytic upcycling of high-density polyethylene via a processive mechanism. *Nat. Catal.* **3**, 893–901 (2020).
14. Lazar, A., Croitoru, C., Tierean, M. & Baltes, L. Thermal and thermorheologic characterization of different polyolefin waste fractions. *Mater. Sci. Forum* **907**, 74–79 (2017).
15. Robert S. Brodkey & Hershey, H. C. Agitation. in *Transport Phenomena: a Unified Approach* 250–268 (McGraw-Hill, New York, USA, 1988).
16. Gedde, U. W. *et al.* Molecular structure, crystallization behavior, and morphology of fractions obtained from an extrusion grade high-density polyethylene. *Polym. Eng. Sci.* **28**, 1289–1303 (1988).

17. Sakai, T. Single polymer chain. in *Physics of Polymer Gels* 1–22 (Wiley‐VCH Verlag GmbH & Co. KGaA, 2020).
18. López Cascales, J. J. & García de la Torre, J. Simulation of polymer chains in elongational flow. Kinetics of chain fracture and fragment distribution. *J. Chem. Phys.* **97**, 4549–4554 (1992).
19. Shinohara, K., Yanagisawa, M. & Makida, Y. Direct observation of long-chain branches in a low-density polyethylene. *Sci. Rep.* **9**, 9791 (2019).
20. Durrill, P. L. & Griskey, R. G. Diffusion and solution of gases into thermally softened or molten polymers: Part II. Relation of diffusivities and solubilities with temperature pressure and structural characteristics. *AIChE J.* **15**, 106–110 (1969).
21. Jaydev, S. D. *et al.* Consumer grade polyethylene recycling via hydrogenolysis on ultrafine supported ruthenium nanoparticles. *Angew. Chem. Int. Ed.* e202317526 (2023).
